# Exogenous methyl jasmonate promotes the graft union healing process of melon grafted onto squash and enhances low-temperature tolerance of grafted melon seedlings by activating the transcription factors CmWRKY23 and CmWRKY69

**DOI:** 10.1093/hr/uhag220

**Published:** 2026-06-03

**Authors:** Yulei Zhu, Hongxi Wu, Jingwei Liu, Yinuo Liang, Xinzhuo Miao, Hongai Liu, Chuanqiang Xu, Tianlai Li

**Affiliations:** College of Horticulture, Shenyang Agricultural University, Shenyang 110866, China; Key Laboratory of Protected Horticulture of Education of Ministry and Liaoning Province, National & Local Joint Engineering Research Center of Northern Horticultural Facilities Design & Application Technology, Shenyang 110866, China; Key Laboratory of Horticultural Equipment, Ministry of Agriculture and Rural Affairs, Shenyang 110866, China; Modern Protected Horticulture Engineering & Technology Center, Shenyang Agricultural University, Shenyang 110866, China; College of Horticulture, Shenyang Agricultural University, Shenyang 110866, China; Key Laboratory of Protected Horticulture of Education of Ministry and Liaoning Province, National & Local Joint Engineering Research Center of Northern Horticultural Facilities Design & Application Technology, Shenyang 110866, China; Key Laboratory of Horticultural Equipment, Ministry of Agriculture and Rural Affairs, Shenyang 110866, China; Modern Protected Horticulture Engineering & Technology Center, Shenyang Agricultural University, Shenyang 110866, China; College of Horticulture, Shenyang Agricultural University, Shenyang 110866, China; Key Laboratory of Protected Horticulture of Education of Ministry and Liaoning Province, National & Local Joint Engineering Research Center of Northern Horticultural Facilities Design & Application Technology, Shenyang 110866, China; Key Laboratory of Horticultural Equipment, Ministry of Agriculture and Rural Affairs, Shenyang 110866, China; Modern Protected Horticulture Engineering & Technology Center, Shenyang Agricultural University, Shenyang 110866, China; College of Horticulture, Shenyang Agricultural University, Shenyang 110866, China; Key Laboratory of Protected Horticulture of Education of Ministry and Liaoning Province, National & Local Joint Engineering Research Center of Northern Horticultural Facilities Design & Application Technology, Shenyang 110866, China; Key Laboratory of Horticultural Equipment, Ministry of Agriculture and Rural Affairs, Shenyang 110866, China; Modern Protected Horticulture Engineering & Technology Center, Shenyang Agricultural University, Shenyang 110866, China; College of Horticulture, Shenyang Agricultural University, Shenyang 110866, China; Key Laboratory of Protected Horticulture of Education of Ministry and Liaoning Province, National & Local Joint Engineering Research Center of Northern Horticultural Facilities Design & Application Technology, Shenyang 110866, China; Key Laboratory of Horticultural Equipment, Ministry of Agriculture and Rural Affairs, Shenyang 110866, China; Modern Protected Horticulture Engineering & Technology Center, Shenyang Agricultural University, Shenyang 110866, China; College of Horticulture, Shenyang Agricultural University, Shenyang 110866, China; Key Laboratory of Protected Horticulture of Education of Ministry and Liaoning Province, National & Local Joint Engineering Research Center of Northern Horticultural Facilities Design & Application Technology, Shenyang 110866, China; Key Laboratory of Horticultural Equipment, Ministry of Agriculture and Rural Affairs, Shenyang 110866, China; Modern Protected Horticulture Engineering & Technology Center, Shenyang Agricultural University, Shenyang 110866, China; College of Horticulture, Shenyang Agricultural University, Shenyang 110866, China; Key Laboratory of Protected Horticulture of Education of Ministry and Liaoning Province, National & Local Joint Engineering Research Center of Northern Horticultural Facilities Design & Application Technology, Shenyang 110866, China; Key Laboratory of Horticultural Equipment, Ministry of Agriculture and Rural Affairs, Shenyang 110866, China; Modern Protected Horticulture Engineering & Technology Center, Shenyang Agricultural University, Shenyang 110866, China; College of Horticulture, Shenyang Agricultural University, Shenyang 110866, China; Key Laboratory of Protected Horticulture of Education of Ministry and Liaoning Province, National & Local Joint Engineering Research Center of Northern Horticultural Facilities Design & Application Technology, Shenyang 110866, China; Key Laboratory of Horticultural Equipment, Ministry of Agriculture and Rural Affairs, Shenyang 110866, China; Modern Protected Horticulture Engineering & Technology Center, Shenyang Agricultural University, Shenyang 110866, China

## Abstract

The involvement of plant hormones in the graft union healing process is significant; yet, how exogenous MeJA affects graft union healing and stress tolerance in cucurbit seedlings remains poorly understood at the molecular level. Exogenous MeJA treatment significantly accelerated the healing process of oriental melon grafted onto squash rootstocks. This treatment led to increased expression of CmWRKY23, CmWRKY69, and the cell adhesion-related gene *CmGH9B3* at the graft union, providing evidence for the participation of the CmWRKY23/CmWRKY69-*CmGH9B3* module in the MeJA-facilitated graft union-healing process. Furthermore, CmWRKY23 and CmWRKY69 function as transcriptional activators by directly binding to the promoter of *CmAOC2*, a key jasmonic acid (JA) biosynthesis gene, thereby upregulating *CmAOC2* expression. Knockout mutants of CmWRKY23 or CmWRKY69 significantly decreased the low-temperature tolerance of grafted oriental melon seedlings, accompanied by decreased antioxidant enzyme (SOD, POD, CAT) activity, downregulated *CmAOC2* expression, and reduced endogenous JA levels. These findings further supported the role of this transcriptional module in regulating low-temperature tolerance through the JA signaling pathway. Overall, our work established that exogenous MeJA promoted graft union healing and enhanced the low-temperature tolerance in grafted oriental melon seedlings. This coordination was achieved via the CmWRKY23/CmWRKY69-*CmGH9B3* module for graft union healing and a CmWRKY23/CmWRKY69-*CmAOC2* positive feedback loop for low-temperature adaptation. This study revealed a dual regulatory mechanism by which MeJA coordinated both graft union healing and low-temperature resistance, providing new theoretical foundations and practical targets for improving the quality and environmental adaptability of grafted oriental melon seedlings.

## Introduction

For growing cucurbit vegetables, grafting represents a common and vital agronomic approach [1]. By combining high-quality scions with rootstocks that possess strong stress tolerance, it can substantially improve crop yield and quality, as well as tolerance to both biotic and abiotic stresses. The efficiency and quality of graft union formation directly determine the survival and subsequent growth performance of grafted seedlings [2, 3]. Therefore, elucidating the regulatory mechanisms that promote rapid and effective union formation between scion and rootstock is of significant theoretical and practical importance.

Cold stress, also known as low temperature, is a significant abiotic stress that causes substantial economic losses for growers worldwide [4]. As a thermophilic species, melon (*Cucumis melo* L.) displays high sensitivity to cold climates [5]. As a widely used agronomic practice in horticultural production, grafting is vital for enhancing the ability of various horticultural crops to resist multiple environmental pressures, such as soil-borne diseases, salt stress, and damage from low temperatures [6]. Previous studies have shown that grafting onto squash rootstocks can improve watermelon’s cold tolerance, with jasmonic acid (JA) playing a crucial role [7]. Recent work has revealed the wound-induced JA signaling pathway as one of the most important regulatory pathways following the cutting of the graft unions. Tomato (*Solanum lycopersicum* L.) research has revealed that wounding triggers rapid synthesis of jasmonic acid and its bioactive derivatives like MeJA, which then mediate graft union healing [8]. Understanding how MeJA influences graft union healing between oriental melon scions and squash rootstocks is critical.

The graft healing process is centrally regulated by plant hormones [9]. Methyl jasmonate (MeJA), a derivative of jasmonic acid, acts as a crucial signaling molecule involved in plant wound responses and defense mechanisms [10]. Numerous studies indicate that jasmonates (JAs) promote the induction of low-temperature tolerance in plants. JA buildup and the expression of its biosynthesis-related genes are quickly triggered by cold stress [11]. Additionally, applying MeJA externally boosts cold tolerance in *Arabidopsis*, whereas mutants lacking JA biosynthesis genes become overly sensitive to low temperature [12]. α-Linolenic acid (α-LeA) has been identified in previous research as the initial substrate in the essential module of JA biosynthesis and transduction. Prior to the conversion of α-LeA into 12-oxophytodienoic acid (OPDA), it is emitted from the plastid membrane via phospholipase A1 (PLA1) and undergoes continuous catalysis by 13-lipoxygenase (LOX), allene oxide synthase (AOS), and allene oxide cyclase (AOC). After reduction, OPDA undergoes three β-oxidation cycles, resulting in JA formation [13]. Most transcription factors (TFs) involved in regulating JA biosynthesis during oriental melon’s cold stress response have not yet been identified.

Organisms rely primarily on transcriptional regulation to modulate gene expression [8]. The WRKY family ranks as one of the largest transcription factor groups unique to higher plants. Usually, WRKY TFs fall into three subfamilies, including clades I, II, and III. WRKY proteins are capable of acting as regulators through their interaction with DNA sequences (C/T) TGAC (T/C), specifically referred to as W-box elements [14]. In the context of cold stress, a variety of TFs have been identified, such as WRKYs, ERFs, and MYBs. These TFs help regulate JA signaling pathways by modulating the expression of biosynthetic and signaling transduction genes, either directly or indirectly [11]. For example, NaWRKY3 could promote JA accumulation by inducing the transcription of genes critical for JA biosynthesis (AOS, LOX, and AOC) [12]. In grapes, VvWRKY5 enhances resistance to grape white rot by regulating the JA pathway [13]. Yet, how JA regulates grafted oriental melon seedlings under low-temperature stress remains largely unknown at the molecular level.

In summary, this study aimed to elucidate the dual regulatory mechanism by which exogenous methyl jasmonate (MeJA) synergistically promoted graft wound healing and enhanced low-temperature tolerance in plants. Our study confirmed that exogenous MeJA activated the CmWRKY23 and CmWRKY69 transcriptional module, which directly upregulated *CmGH9B3*, thereby facilitating cell adhesion and healing at the graft interface. Concurrently, this module induced *CmAOC2* expression, establishing a positive feedback loop that increased endogenous jasmonic acid (JA) biosynthesis and activated downstream defense responses, thereby coordinating graft union healing and stress adaptation. These findings not only elucidated the molecular network underlying the synergistic regulation of MeJA-mediated graft union healing and low-temperature tolerance but also provided new theoretical foundations and practical objectives for improving the quality and environmental adaptability of grafted oriental melon seedlings.

## Results

### Exogenous MeJA promoted graft union healing process in oriental melon scions through the CmWRKY23/ CmWRKY69-*CmGH9B3* module

In our prior investigations, PlantCARE was employed to predict *cis*-regulatory elements in the *CmGH9B3* promoter, and a MeJA-responsive element was subsequently found among them [15]. Acidic fuchsin staining showed that the vascular connectivity of oriental melon scions treated with exogenous MeJA increased compared with the control (Fig. 1A). Following paraffin section assays, it was observed that under exogenous MeJA treatment, healing tissues formed at 5 DAG, and vascular bundles were attached at 8 DAG. In comparison, the control group experienced a 1-day delay (Fig. 1B).

**Figure 1:**
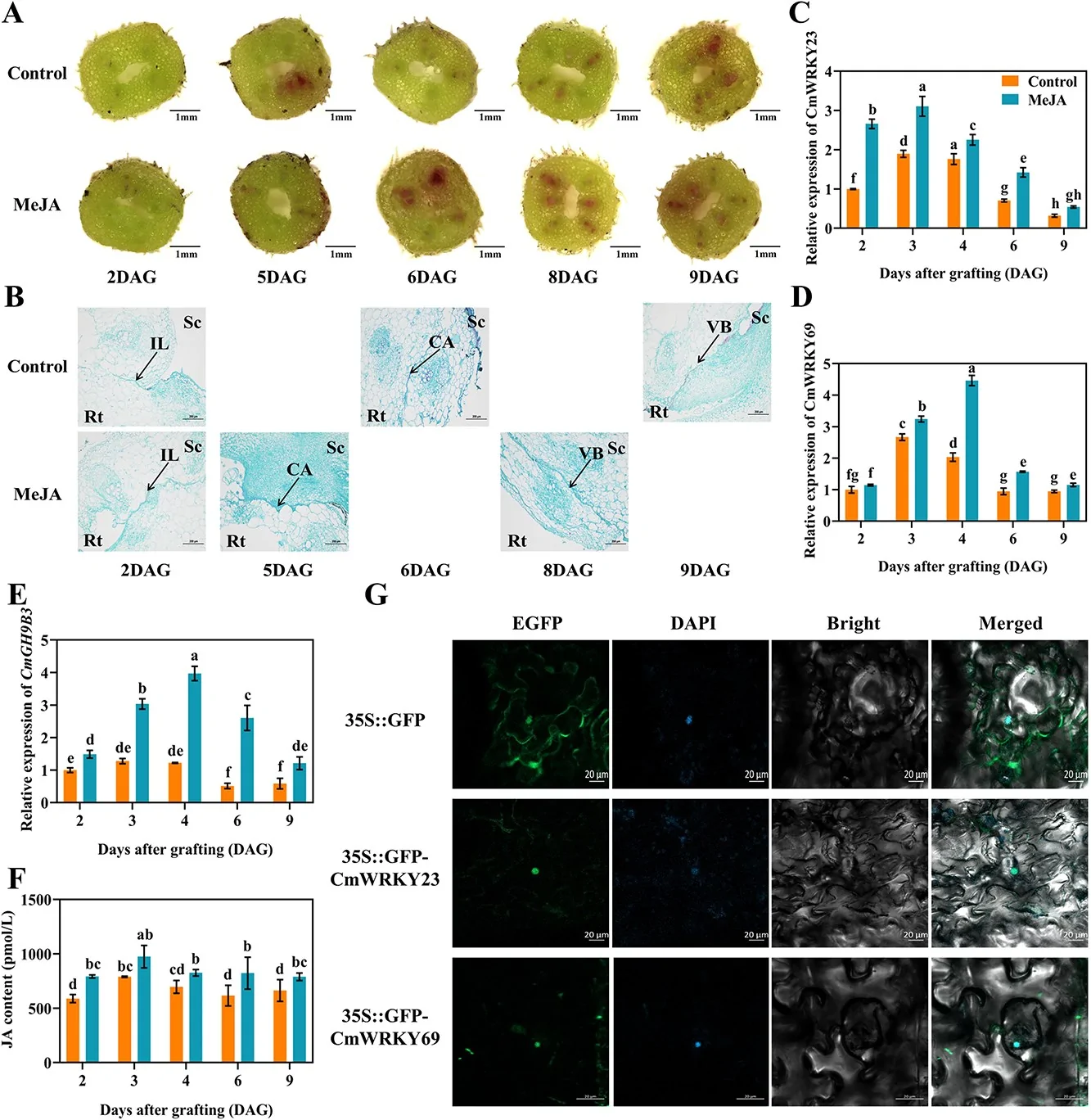
Exogenous MeJA promoted the graft union healing process in oriental melon scions grafted onto squash rootstocks. (A) Acid fuchsin absorption assay in exogenous MeJA treatment and control grafted seedlings at 5–9 days after grafting (DAG). Scale bar = 1 mm. (B) Toluidine-stained sections showing graft interface structure in MeJA and control plants at 2, 5, 6, 8, and 9 DAG (IL, the isolated layer; CA, the callus; VB, the vascular bundles; Sc, scion; Rt, rootstock; MeJA, methyl jasmonate; DAG, days after grafting; scale bar = 200 μm). (C–E) Relative expression analysis of CmWRKY23, CmWRKY69, and *CmGH9B3* in MeJA treatment during graft healing. (F) Effects of exogenous MeJA treatment on endogenous JA content. (G) Subcellular localization of CmWRKY23-GFP and CmWRKY69-GFP fusion protein in *N. benthamiana* epidermal cells. GFP signal was nuclear-localized, and 4′,6-diamidino-2-phenylindole (DAPI) was used to stain the nucleus. Scale bar = 20 μm. Data represent mean ± SD (*n* = 3). Different letters indicate significant differences (ANOVA, *P* < 0.05).

Furthermore, to gain deeper insights into the impacts of exogenous MeJA treatment, we detected transcript levels of healing-related genes at the graft junction, namely CmWRKY23, CmWRKY69, and *CmGH9B3*. During the graft healing process, exogenous MeJA application significantly upregulated transcript levels of CmWRKY23, CmWRKY69, and *CmGH9B3*, accompanied by elevated endogenous jasmonic acid (JA) levels relative to the control group (Fig. 1C–F). Utilizing *Agrobacterium*-mediated transient transformation, we infiltrated *Nicotiana benthamiana* leaves to ascertain the subcellular distribution of CmWRKY23 and CmWRKY69, revealing their presence in the nucleus of *N. benthamiana* cells (Fig. 1G).

Then, for a deeper exploration of the roles of CmWRKY23 and CmWRKY69 in graft union formation, ‘T0948’ functioned as the wild type (WT), and single as well as double knockout mutants of *Cmwrky23* and *Cmwrky69* were generated (Fig. 2A). As shown in Fig. 2B, phenotypic analysis of grafted seedlings revealed no obvious morphological differences among WT, *Cmwrky23,* and *Cmwrky69* mutants. However, compared with WT, these mutants displayed delayed acid fuchsin staining during graft union healing process (Fig. 2C). Expression levels of *CmGH9B3* were significantly reduced (Fig. 2D–H), accompanied by decreased β-1,4-glucanase activity, which was more pronounced in the double knockout mutant (Fig. 2I). Exogenous MeJA exhibited a beneficial effect on graft union healing in oriental melon scions grafted onto squash rootstocks, whereas the knockout of CmWRKY23 and CmWRKY69 impeded this restorative process.

**Figure 2:**
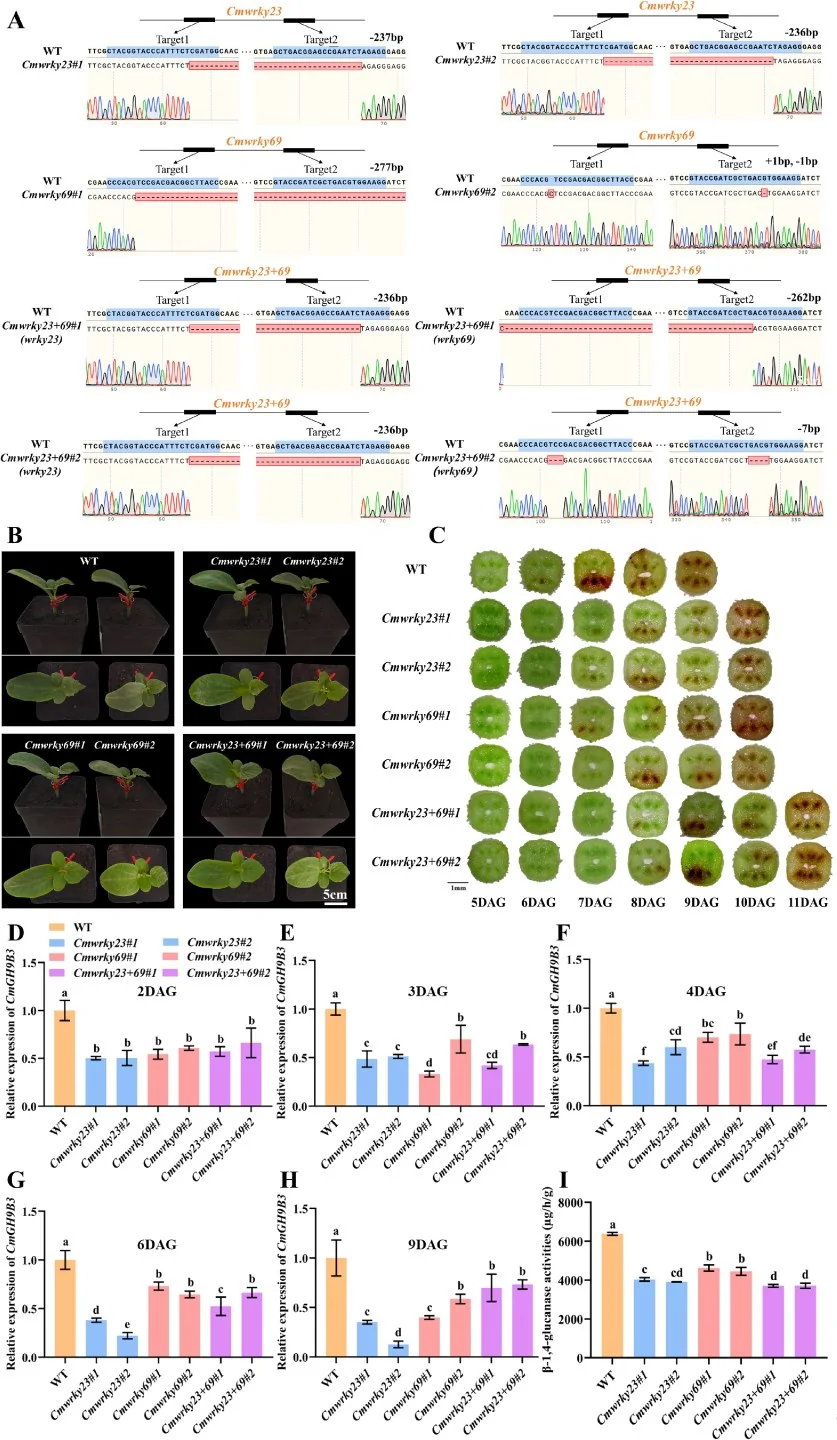
CmWRKY23 and CmWRKY69 knockouts repressed the graft union healing process in oriental melon scions grafted onto squash rootstocks. (A) Identification of CmWRKY23-knockout and CmWRKY69-knockout plants. Gene-edited sites of the *Cmwrky23* (#1, #2), *Cmwrky69* (#1, #2), and *Cmwrky23*+*69* (#1, #2) mutants. (B) Phenotypes of WT and transgenic oriental melon grafted seedlings. Scale bars, 5 cm. (C) Acid fuchsin absorption assay in WT and *Cmwrky23*, *Cmwrky69* mutant grafted seedlings. Scale bar = 1 mm. (D–H) Expression analysis of *CmGH9B3* among the WT and *Cmwrky23*, *Cmwrky69* mutant grafted seedlings. WT, wild-type grafted seedlings. (I) The activities of β-1,4-glucanase in WT and *Cmwrky23*, *Cmwrky69* mutant grafted seedlings. Data represent mean ± SD (*n* = 3). Different letters indicate significant differences (ANOVA, *P* < 0.05).

### Expression profiles of genes related to JA biosynthesis in oriental melon scion under exogenous MeJA treatment during the graft union healing process

For the purpose of investigating the effects of exogenous MeJA on genes associated with JA biosynthesis, we assessed the expression patterns of five key JA synthesis pathway genes (AOC2, JAR1, AOS, OPR3, and LOX2) at different stages of graft union healing (Fig. 3). As graft union healing progressed, there was a gradual increase in the relative expression of *CmAOC2*. It was remarkably upregulated under the application of exogenous MeJA (Fig. 3A). Exogenous MeJA treatment did not raise the transcript levels of *CmJAR1* or *CmAOS* (Fig. 3B and C). The relative expression of *CmOPR3* was significantly elevated only at 4 and 6 DAG (Fig. 3D). Only at 4 DAG did the relative expression of *CmLOX2* show a significant increase (Fig. 3E). It was evident that exogenous MeJA treatment had variable effects on the expression of key genes involved in JA biosynthesis during graft union healing, with the most pronounced induction observed in the *CmAOC2* gene.

**Figure 3:**
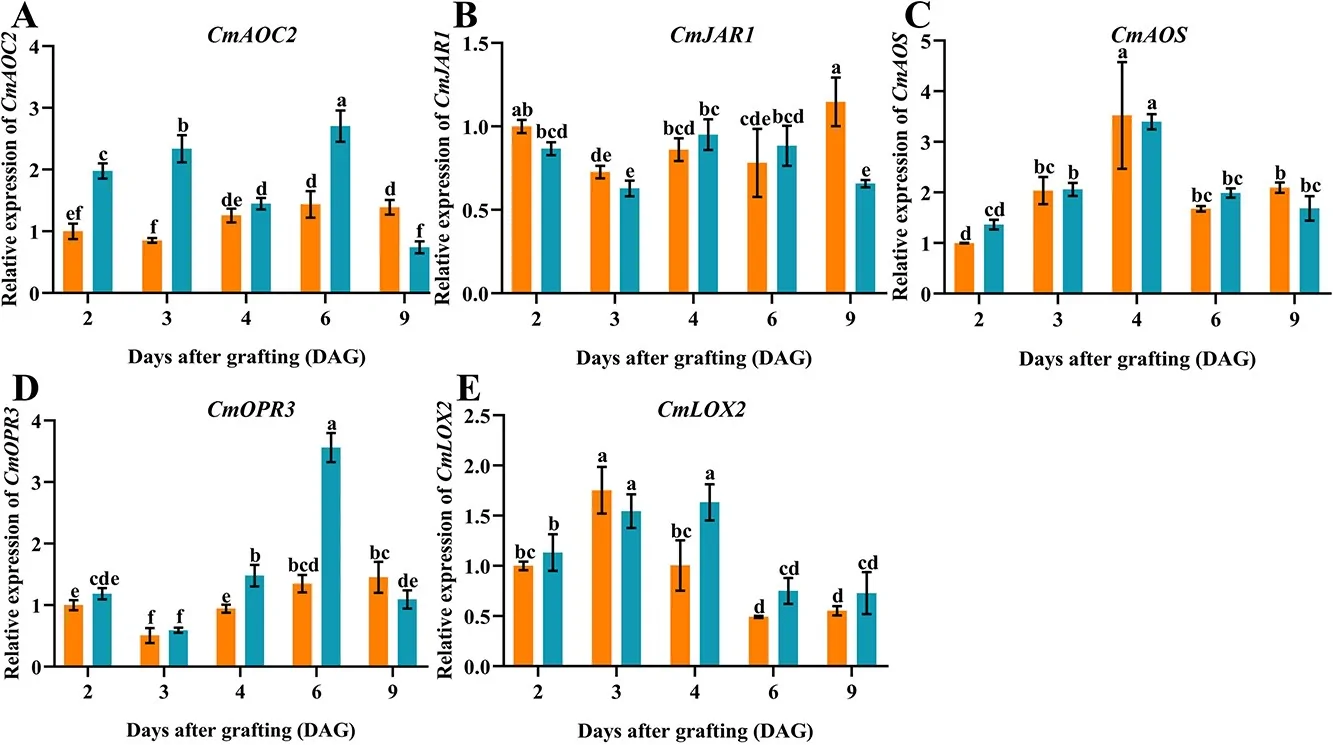
Effect of exogenous MeJA treatment on key genes for JA biosynthesis in oriental melon scion. (A) *CmAOC2*. (B) *CmJAR1*. (C) *CmAOS*. (D) *CmOPR3*. (E) *CmLOX2*. DAG, days after grafting. Data represent mean ± SD (*n* = 3). Different letters indicate significant differences (ANOVA, *P* < 0.05).

### Exogenous MeJA treatment enhanced the low-temperature tolerance of grafted oriental melon seedlings

JA is a key hormone that regulates plants’ responses to low-temperature stimuli [16]. To determine how JA affects the low-temperature response of grafted oriental melon seedlings, we first examined whether exogenous MeJA and DIECA (an inhibitor of JA biosynthesis) treatments affected grafted seedlings’ low-temperature tolerance. Visible damage symptoms emerged following low-temperature exposure (e.g., leaf drooping and wilting) in the treated plants. The MeJA (200 μM) treatment alleviated these low-temperature-induced symptoms, whereas DIECA exacerbated them (Fig. 4A). Diaminobenzidine (DAB) staining revealed brown spots within leaves of oriental melon seedlings under 4°C low-temperature stress, compared with normal plants. There were fewer brown spots after exogenous MeJA treatment, whereas the area of brown spots increased significantly after DIECA treatment (Fig. 4B). The results of nitroblue tetrazolium (NBT) staining were also similar (Fig. 4C).

**Figure 4:**
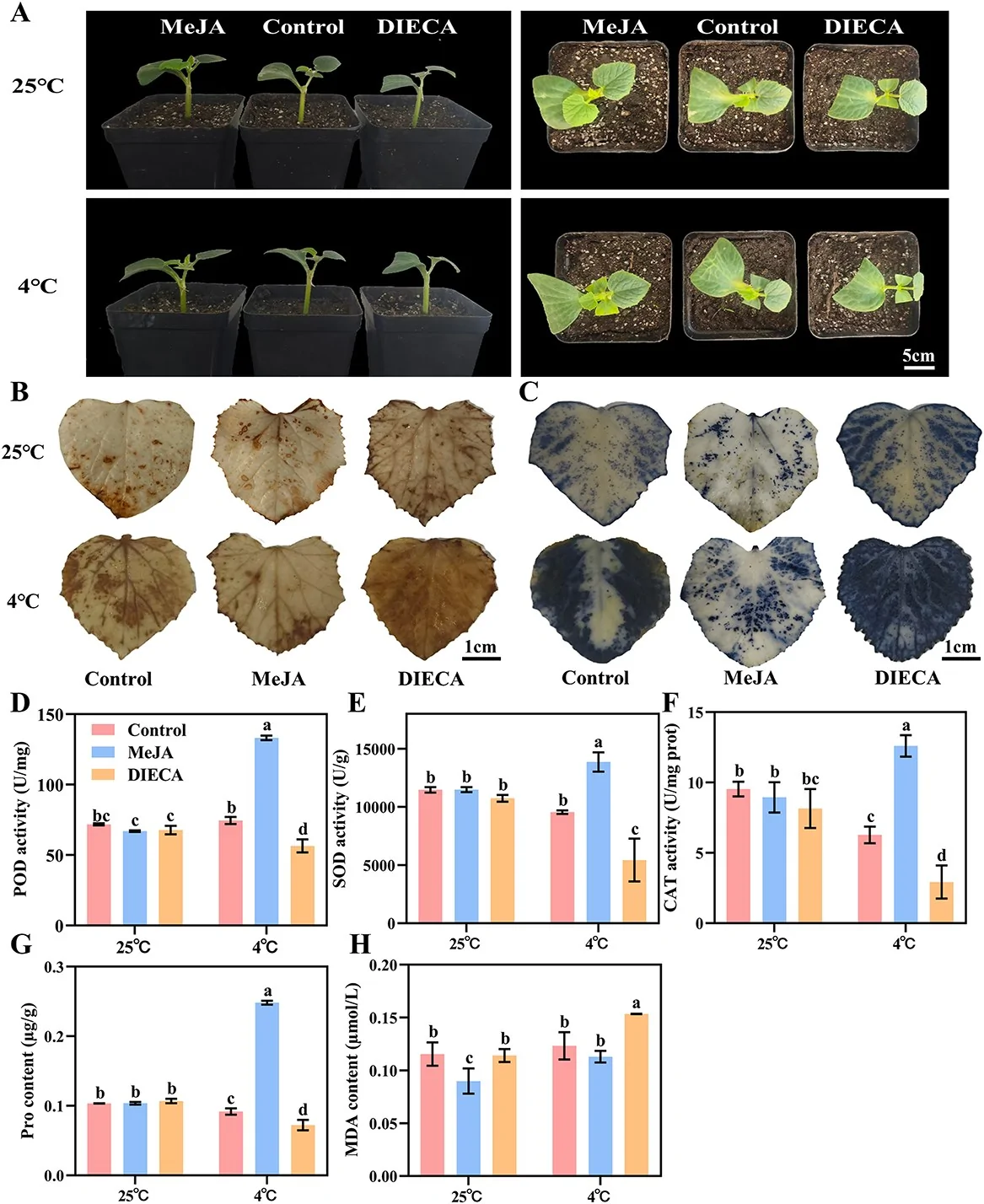
Effect of exogenous MeJA and DIECA treatments on low-temperature tolerance of the grafted oriental melon seedlings. (A) Plant phenotypes. Scale bar, 5 cm (B) DAB staining. (C) NBT staining. Scale bar = 1 cm. (D) POD enzyme activity. (E) SOD enzyme activity. (F) CAT enzyme activity. (G) Pro content. (H) MDA content. MeJA, methyl jasmonate; DIECA, diethyldithiocarbamic acid (JA biosynthesis inhibitor). Data represent mean ± SD (*n* = 3). Different letters indicate significant differences (ANOVA, *P* < 0.05).

The antioxidant system primarily regulates lipid peroxidation in plants [7]. As shown in Fig. 4D–F, POD, SOD, and CAT (peroxidase, superoxide dismutase, and catalase) activities showed no notable variation between 4°C-grown seedlings and those under normal temperature. However, under 4°C low-temperature stress, the activities of POD, SOD, and CAT were significantly higher in seedlings treated with exogenous MeJA than in the control plants, whereas DIECA-treated seedlings showed the opposite trend.

To assess how JA affects the physiological responses of oriental melon grafted seedlings under low-temperature stress, several indicators were examined, including proline (Pro) content and malondialdehyde (MDA) levels. Our data demonstrated that MeJA-treated oriental melon scion grafted onto squash seedlings exhibited substantially greater Pro content than control plants under low-temperature stress, whereas DIECA reduced Pro content by a marked degree (Fig. 4G). However, MDA metabolism levels obtained the opposite result (Fig. 4H). The results collectively indicate that MeJA treatment improved low-temperature tolerance in grafted oriental melon seedlings.

### Analysis of differential expression of *CmAOC2* in oriental melon seedlings

As shown in the above study, exogenous MeJA notably induced the upregulation of *CmAOC2*, a key gene in JA biosynthesis, in the grafted oriental melon seedlings. Analysis of *CmAOC2* expression in four tissues of oriental melon indicated a primary expression in the root, stems, stem tips, and leaves, as shown in Fig. 5A.

**Figure 5:**
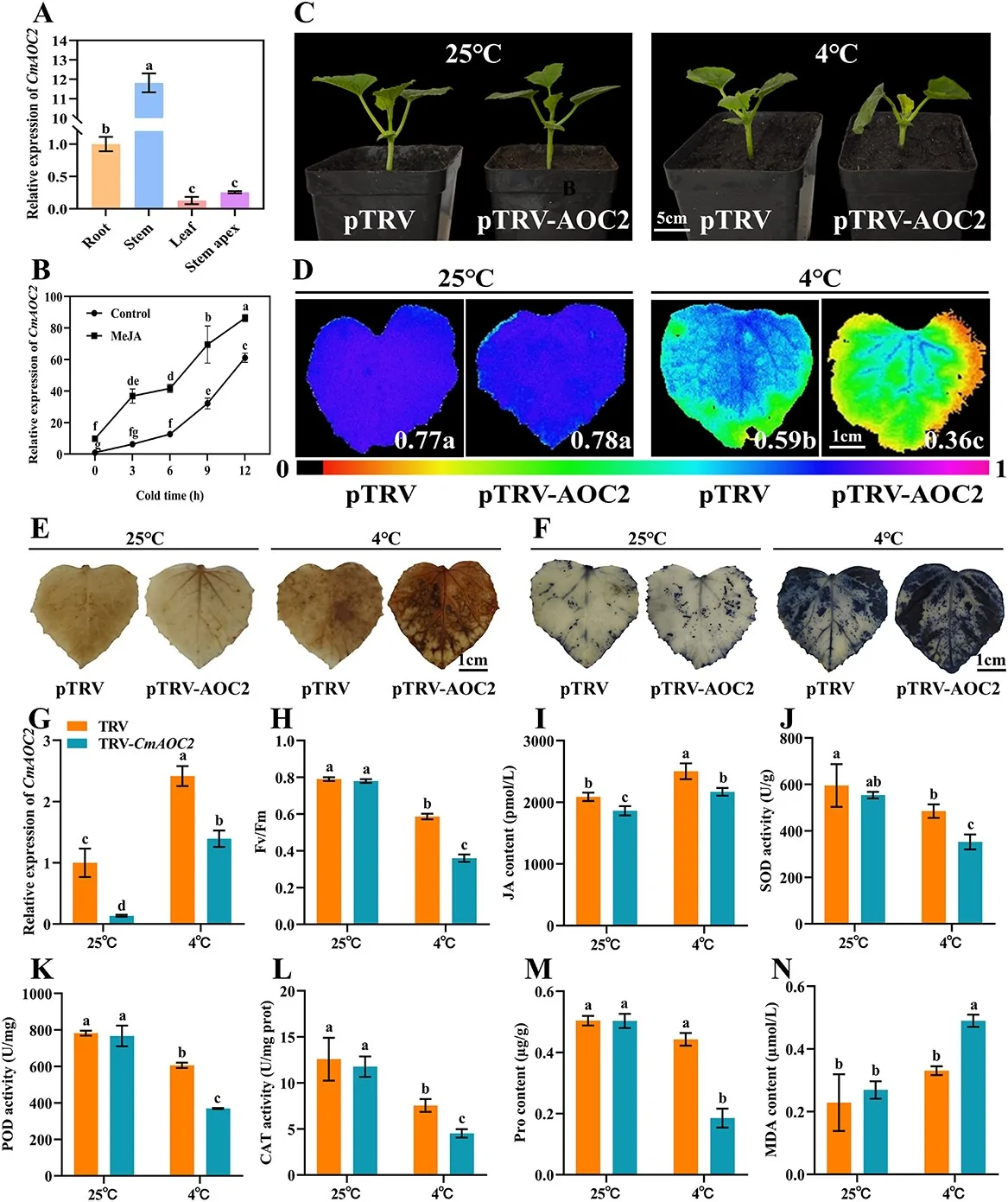
Silencing *CmAOC2* reduced the low-temperature sensitivity of oriental melon seedlings. (A) Relative expression of *CmAOC2* in 4 tissues of roots, stems, leaves, and stem tips in oriental melon, with the expression of roots set as 1. (B) RT-qPCR was used to analyze the *CmAOC2* expression level in leaf tissues under different low-temperature treatment durations. (C) Plant phenotype. Scale bar, 5 cm. (D) Images of the maximum photochemical efficiency of PSII (Fv/Fm). The intensity scale at the bottom of the image ranges from 0 to 1. Different letters indicate significant differences at *P* < 0.05 (one-way ANOVA). Scale bar = 1 cm. (E) DAB staining. (F) NBT staining. Scale bar = 1 cm. (G) Expression level of *CmAOC2* in the silenced plants under low-temperature stress. (H) Fv/Fm ratio. (I) Endogenous JA content. (J) SOD activity. (K) POD activity. (L) CAT activity. (M) Pro activity. (N) MDA content. Data represent mean ± SD (*n* = 3). Different letters indicate significant differences (ANOVA, *P* < 0.05).

After exogenous MeJA treatment, we subjected oriental melon autogenous seedlings to different durations of low-temperature treatment and conducted a more comprehensive RT-qPCR analysis of *CmAOC2* expression in seedling leaves. The results showed that *CmAOC2* expression rose gradually over the initial 9 h and was significantly induced between 9 and 12 h in control plants. At all time points, transcript abundance in plants receiving exogenous MeJA treatment was markedly higher than in control plants (Fig. 5B). Following this preliminary expression analysis, it is conjectured that *CmAOC2* may function as a potential regulator of oriental melon low-temperature tolerance.

To explore *CmAOC2*’s function in the low-temperature tolerance of oriental melon seedlings, we have chosen to assess the low-temperature tolerance of *CmAOC2*-silenced plants (pTRV-*CmAOC2*). In comparison with the pTRV control plants, the pTRV-*CmAOC2* plants exhibited severe wilting and chilling-induced damage after being subjected to 48 h of low-temperature treatment (Fig. 5C). Prior to low-temperature treatment, pTRV-*CmAOC2* plants showed comparable Fv/Fm values to control plants. Nevertheless, after low-temperature treatment, a sharp drop in Fv/Fm occurred in pTRV-*CmAOC2* (Fig. 5D and H). DAB and NBT staining revealed that TRV-*CmAOC2* plants are affected by 4°C low-temperature stress (Fig. 5E and F).

In addition, under both normal and low temperatures, *CmAOC2* expression was reduced in pTRV-*CmAOC2* plants relative to WT (Fig. 5G). There was also a significant reduction in endogenous JA content, and the difference became more marked at low temperature (Fig. 5I). pTRV-*CmAOC2* plants grown at 4°C exhibited significantly inhibited activities of the antioxidant enzymes SOD, POD, and CAT, together with reduced proline content (Pro), compared with pTRV plants (Fig. 5J–M). The results for MDA content were opposite (Fig. 5N).

We also generated *CmAOC2*-OE lines and examined their physiological responses, as well as those of WT plants, following 48 h of cold exposure. As seen in Fig. S1B, severe stress was displayed by WT plants, whereas *CmAOC2*-OE plants were affected to a lesser extent. For instance, under normal growth conditions, WT and *CmAOC2*-OE plants showed similar Fv/Fm values. However, under low-temperature stress, Fv/Fm declined to 0.56 in WT versus 0.62 in *CmAOC2*-OE (Fig. S1A and D). *CmAOC2* transcript levels rose markedly as well, with an even more pronounced difference under low-temperature stress (Fig. S1C). Furthermore, endogenous JA content yielded the same results (Fig. S1E). In addition, when subjected to low-temperature stress, the activities of antioxidant enzymes (SOD, POD, and CAT) and Pro content in *CmAOC2*-OE seedlings were significantly higher compared to WT plants (Fig. S1F–I). MDA data further confirmed that *CmAOC2*-OE plants tolerated low-temperature stress better than WT plants (Fig. S1J).

### CmWRKY23 and CmWRKY69 directly bound to the promoter of *CmAOC2* to activate their expression

We conducted *cis*-acting element analysis of promoter sequences situated within the region located 2.0 kb upstream of the transcription initiation site of *CmAOC2* to determine the primary transcription factors responsible for the increased expression of *CmAOC2* at low temperature. The W-box (C/TTGACT/C), a WRKY-binding element, as well as its core TGAC motif, were detected in the promoter regions (Fig. 6). As confirmed in our previous studies, red light promotes graft healing efficiency by means of the CmHY5-CmWRKY23/CmWRKY69-*CmGH9B3* module [17]. In this study, exogenous MeJA treatment can also induce the CmWRKY23/CmWRKY69-*CmGH9B3* module to positively and significantly regulate graft union healing and to upregulate the expression of the key JA biosynthesis factor *CmAOC2*. Therefore, we investigated whether *CmAOC2* improves the low-temperature tolerance in grafted oriental melon seedlings, with this effect dependent on CmWRKY23 and CmWRKY69.

**Figure 6:**
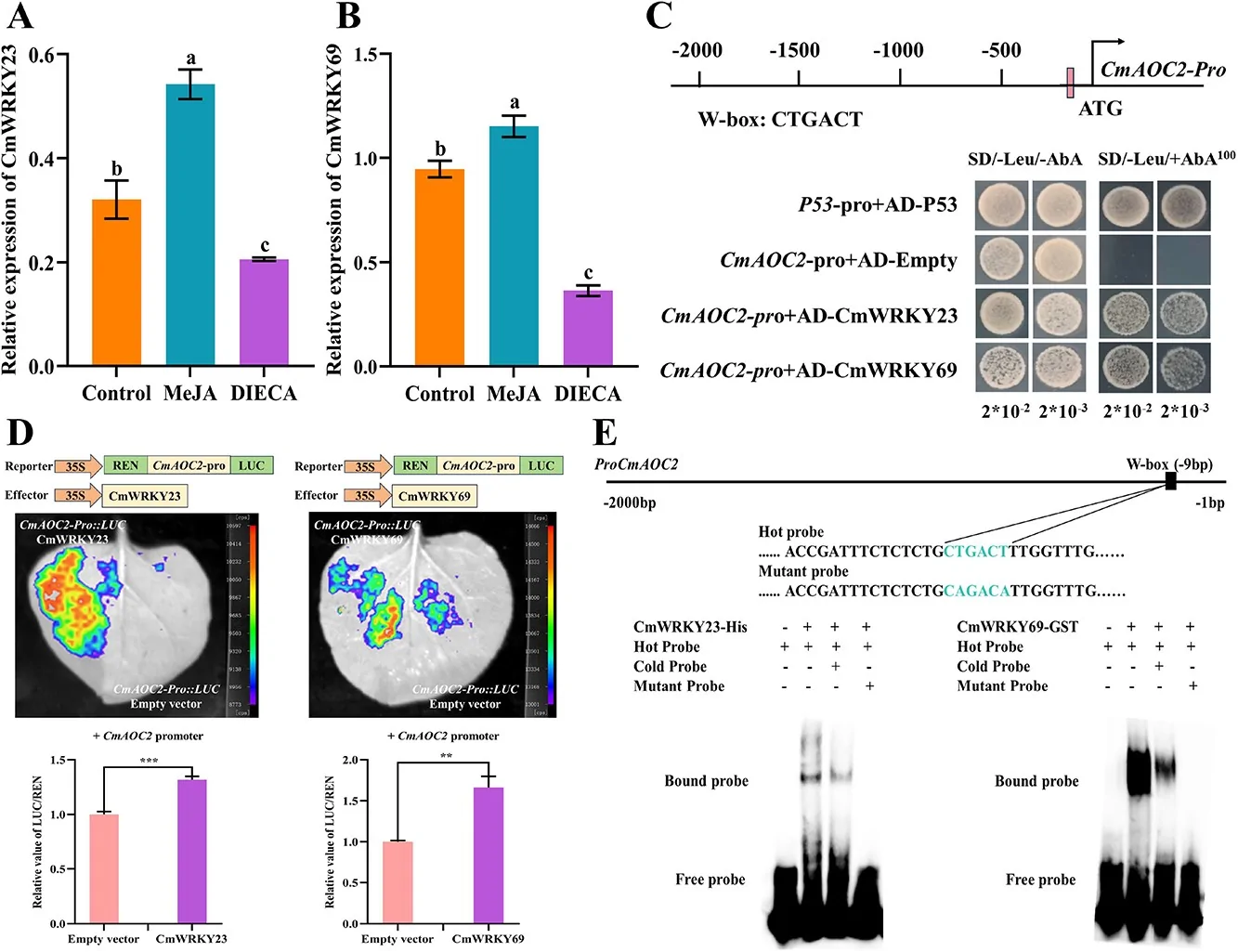
CmWRKY23 and CmWRKY69 bind directly to the promoters of *CmAOC2* as transcriptional activators. (A and B) Relative expression of CmWRKY23 and CmWRKY69 in oriental melon leaves after treatment with exogenous MeJA and DIECA. (C) Y1H analysis of CmWRKY23 and CmWRKY69 binding to the promoter of *CmAOC2*. The growth status of transformed yeasts on two media. Normal yeast growth on a defective medium containing the antibiotic Aureobasidin A indicates that CmWRKY23 and CmWRKY69 can bind to the promoter of *CmAOC2* independently. (D) A Luciferase reporter assay showed that CmWRKY23 and CmWRKY69 bind to the *CmAOC2* promoter *in vivo*. The infected *N. benthamiana* was measured by a living fluorescence imager. The fluorescence intensity exceeds that of the control (empty vector), indicating that CmWRKY23 and CmWRKY69 interact with the promoter and enhance gene expression. (E) EMSA analysis of CmWRKY23 and CmWRKY69 binding to the *CmAOC2* promoter. The hot probe was a biotin-labeled *CmAOC2* promoter, while the cold probe was an unlabeled competitive probe. His-tagged CmWRKY23 and GST-tagged CmWRKY69 (CmWRKY23-His and CmWRKY23-GST) were purified and used for DNA-binding assays. The sequence of the biotin-labeled probe is shown, and the CTGACT motifs are highlighted in bold. The data are the means of three replicates (±SD) (*n* = 3). Statistical significance levels (Student’s *t*-test) are shown: ns; ^*^P ≤ 0.05; ^**^P ≤ 0.01; ^***^P ≤ 0.001; ^****^P ≤ 0.0001.

Examination of gene expression indicated significant upregulation of CmWRKY23 and CmWRKY69 in oriental melon scions following exogenous MeJA treatment, whereas treatment with the inhibitor DIECA showed a contrasting trend (Fig. 6A and B). Therefore, we further confirmed its interaction properties with *CmAOC2*. The roles of CmWRKY23 and CmWRKY69 in gene activation were detected using a yeast one-hybrid (Y1H) assay. During the assay, it was observed that yeast cells harboring pABAi-*CmAOC2*, pGADT7-CmWRKY23, and pGADT7-CmWRKY69 as bait and prey, respectively, thrived on the selective medium. By contrast, yeast growth was much lower on plates harboring the empty vector (Fig. 6C).

To better confirm how CmWRKY23 and CmWRKY69 function in activating genes *in vivo*, we constructed firefly luciferase (LUC) reporter gene vectors controlled by the *CmAOC2* promoter, as well as effector vectors harboring CmWRKY23 and CmWRKY69. Transient expression assays showed that co-expressing effector vectors with reporter genes strongly boosted LUC activity relative to controls, leading to an elevated LUC/*Renilla* ratio in *N. benthamiana* leaves (Fig. 6D). The results were further confirmed by GUS staining detection of *N. benthamiana* plants transiently transformed, demonstrating the activation of GUS reporter genes driven by the *CmAOC2* promoter in response to the effectors CmWRKY23 and CmWRKY69 (Fig. S2A and B).

To determine if CmWRKY23 and CmWRKY69 are capable of directly interacting with the W-box motifs in the *CmAOC2* promoter, an *in vitro* electrophoretic mobility shift assay (EMSA) experiment was conducted. We designed the probes based on the W-box elements in the *CmAOC2* promoter. When His-CmWRKY23 and GST-CmWRKY69 proteins were incubated with the labeled probes, changes in mobility were observed, while adding unlabeled probe competitor DNA led to a dose-dependent decline in electrophoretic mobility shift. When mutated probes were introduced, no protein–DNA complexes formed with CmWRKY23 or CmWRKY69, verifying that both transcription factors directly and specifically target the W-box motifs in the *CmAOC2* promoter (Fig. 6E). Our results indicate that both CmWRKY23 and CmWRKY69 act upstream of *CmAOC2* and stimulate its expression.

### CmWRKY23 and CmWRKY69 were positive factors for oriental melon low-temperature tolerance

For the purpose of clarifying the function of CmWRKY23 and CmWRKY69 in the low-temperature resistance of oriental melon seedlings, we opted for CmWRKY23 and CmWRKY69 silenced plants (pTRV-CmWRKY23, pTRV-CmWRKY69, and pTRV-CmWRKY23+69), with CmWRKY23 and CmWRKY69 transcript levels at only 30% to 40% of those in empty vector plants during low-temperature tolerance assessment (Fig. S3A and B). Relative to pTRV plants, pTRV-CmWRKY23, pTRV-CmWRKY69, and pTRV-CmWRKY23+69 displayed wilting and chilling-induced lesions after 48-h cold treatment (Fig. 7A), in agreement with the physiological findings, including plant height, stem thickness, node spacing, aboveground dry weight, aboveground fresh weight, belowground dry weight, and belowground fresh weight (Fig. S3C–I).

**Figure 7:**
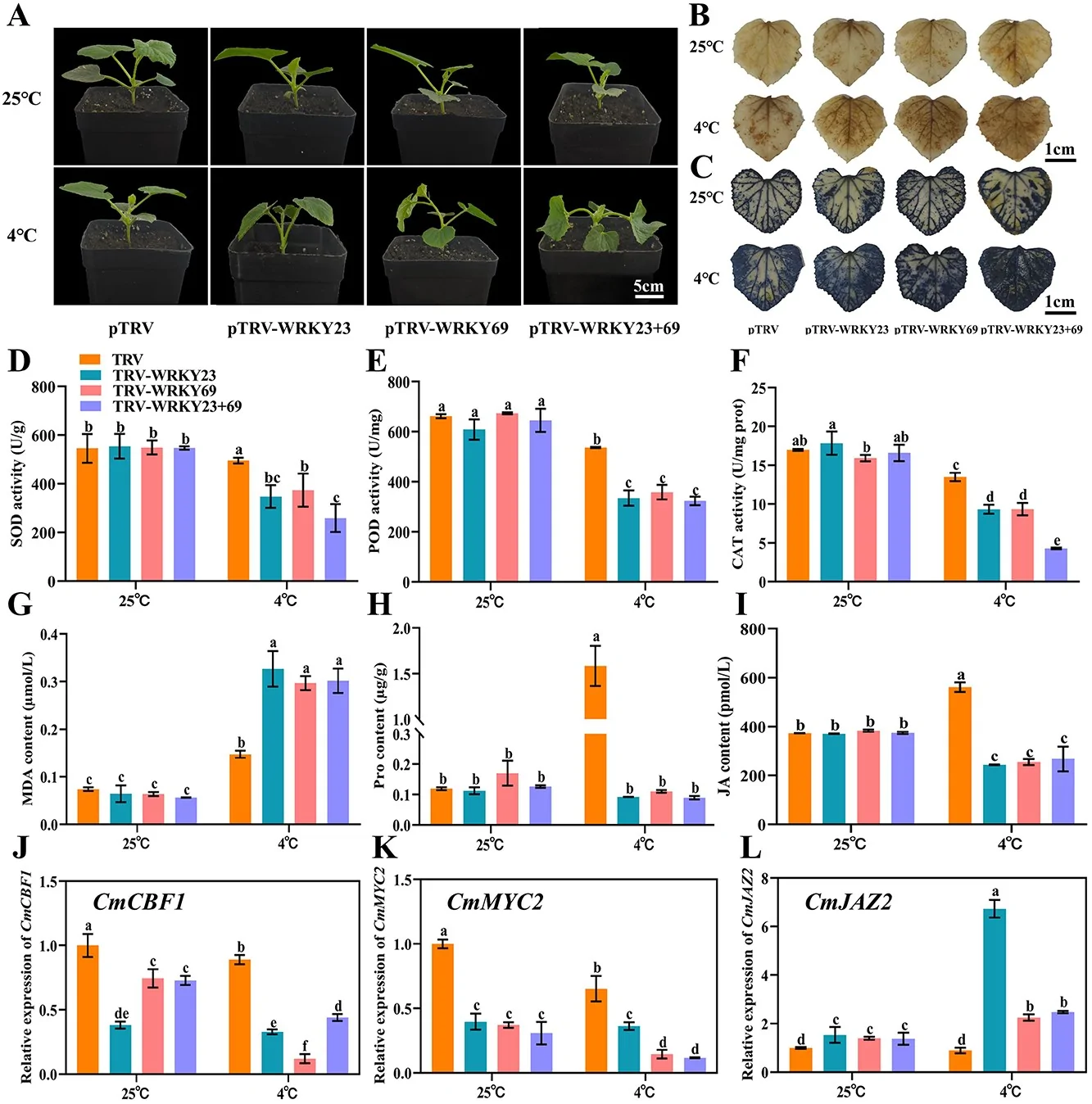
Silencing of CmWRKY23 and CmWRKY69 genes increased low-temperature sensitivity in oriental melon. (A) Plant phenotypes. Scale bar, 5 cm. (B and C) DAB and NBT staining. Scale bar = 1 cm. (D–F) SOD, POD, and CAT enzyme activity. (G–I) MDA, Pro, and JA content. (J–l) Relative expression level of *CmCBF1*, *CmMYC2*, and *CmJAZ2* following transient silencing. Data represent mean ± SD (*n* = 3). Different letters indicate significant differences (ANOVA, *P* < 0.05).

Diaminobenzidine (DAB) staining revealed no significant changes in pTRV-CmWRKY23, pTRV-CmWRKY69, and pTRV-CmWRKY23+69 compared to pTRV plants under normal temperature conditions. In contrast, under low-temperature stress, brown spots were produced in all leaves of oriental melon seedlings. The changes of pTRV-CmWRKY23+69 were more significant (Fig. 7B). The results of NBT staining were also similar (Fig. 7C). As shown in Fig. 7D–F, relative to pTRV plants, the activities of antioxidant enzymes (SOD, POD, and CAT) in pTRV-CmWRKY23, pTRV-CmWRKY69, and pTRV-CmWRKY23+69 plants cultivated at 4°C were significantly downregulated. The contents of MDA, Pro, and JA also showed the same trend, consistent with the above results (Fig. 7G–I).

Notably, we selected the low-temperature-responsive gene *CmCBF1* (MELO3C006869), which was suppressed under low-temperature stress in pTRV-CmWRKY23, pTRV-CmWRKY69, and pTRV-CmWRKY23+69 plants (Fig. 7J). We next examined the expression patterns of *CmMYC2* and *CmJAZ2*, two key genes involved in the JA signaling pathway, and found that *CmMYC2* transcription was suppressed under low-temperature stress. In contrast, *CmJAZ2* transcription was significantly activated by low-temperature stress (Fig. 7K and L).

Transient lines (CmWRKY23-OE, CmWRKY69-OE, CmWRKY23+69-OE) were established, and their overexpression was validated by RT-qPCR, where CmWRKY23 and CmWRKY69 were expressed at significantly higher levels than WT plants under normal temperature conditions, and were also significantly induced under low-temperature treatment (Fig. S4A and B). The expression levels of the *CmAOC2* gene also yielded similar results (Fig. S4C). As indicated in Fig. S4D, WT plants showed severe stress responses, whereas CmWRKY23-OE, CmWRKY69-OE, and CmWRKY23+69-OE plants were relatively less affected. However, under low-temperature stress, Fv/Fm values dropped to 0.56 for WT, 0.64 for CmWRKY23-OE, 0.64 for CmWRKY69-OE, and 0.70 for CmWRKY23+69-OE (Fig. S4E). DAB and NBT staining revealed that the overexpressing plants mitigated low-temperature-induced leaf damage (Fig. S4F and G). Additionally, under low-temperature treatment, OE lines of CmWRKY23, CmWRKY69, and their combination showed markedly higher endogenous JA levels and activities of SOD, POD, and CAT, as well as proline content, compared to WT plants (Fig. S4H–L). MDA data further confirmed that OE lines tolerated low-temperature stress better than WT plants (Fig. S4M).

Next, we utilized the successfully generated CmWRKY23 and CmWRKY69 knockout lines to validate their low-temperature tolerance. Plants of *Cmwrky23* (#1, #2), *Cmwrky69* (#1, #2), and *Cmwrky23*+*69* (#1, #2) exhibited more pronounced wilting and drooping of leaves compared to the WT and showed increased sensitivity to low-temperature stress (Fig. 8A). DAB and NBT staining revealed increased spots on leaves of the knockout lines under low temperatures, indicating accumulation of these compounds in these leaves (Fig. 8B and C). Compared with WT, the SOD, POD, and CAT activities of the knockout plants grown at 4°C were all suppressed (Fig. 8D–F). Additionally, MDA content increased, while proline and endogenous JA content both decreased significantly (Fig. 8G–I). The above results indicate that CmWRKY23 and CmWRKY69 are positive regulators of low-temperature tolerance in oriental melon seedlings.

**Figure 8:**
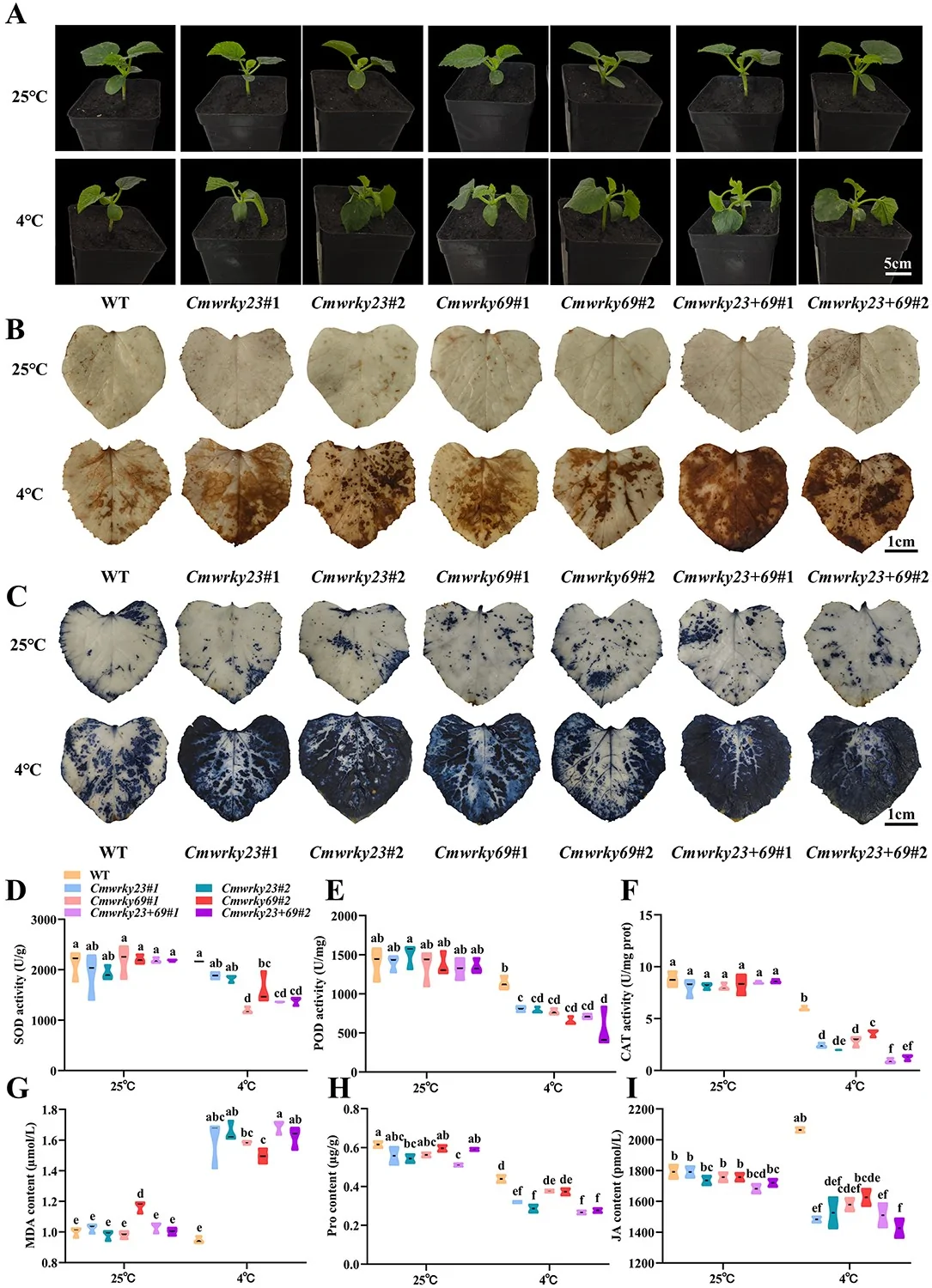
CmWRKY23 and CmWRKY69 knockouts increased low-temperature sensitivity in oriental melon. (A) Plant phenotypes. Scale bar, 5 cm. (B and C) DAB and NBT staining. Scale bar = 1 cm. (D–F) SOD, POD, and CAT enzyme activity. (G–I) Pro, MDA, and JA content. Data represent mean ± SD (*n* = 3). Different letters indicate significant differences (ANOVA, *P* < 0.05).

### CmWRKY23 and CmWRKY69 modulated the low-temperature tolerance by directly activating JA signaling

To better understand whether CmWRKY23 and CmWRKY69 positively regulate low-temperature tolerance by JA accumulation, we applied MeJA to *Cmwrky23*#1, *Cmwrky69*#1, and *Cmwrky23*+*69*#1 plants, with ddH_2_O sprayed as a control. In Fig. 9A, knockout plants treated with MeJA showed a higher Fv/Fm under low-temperature stress than untreated ones (Fig. 9D). The leaves of knockout plants sprayed with MeJA exhibited enhanced low-temperature resistance under low-temperature stress, as reflected by DAB and NBT staining, antioxidant enzyme activities (SOD, POD, CAT), and the accumulation of proline and MDA (Fig. 9B, C, and  E–I). These results reinforced the notion that JA is vital to plant survival under low-temperature conditions.

**Figure 9:**
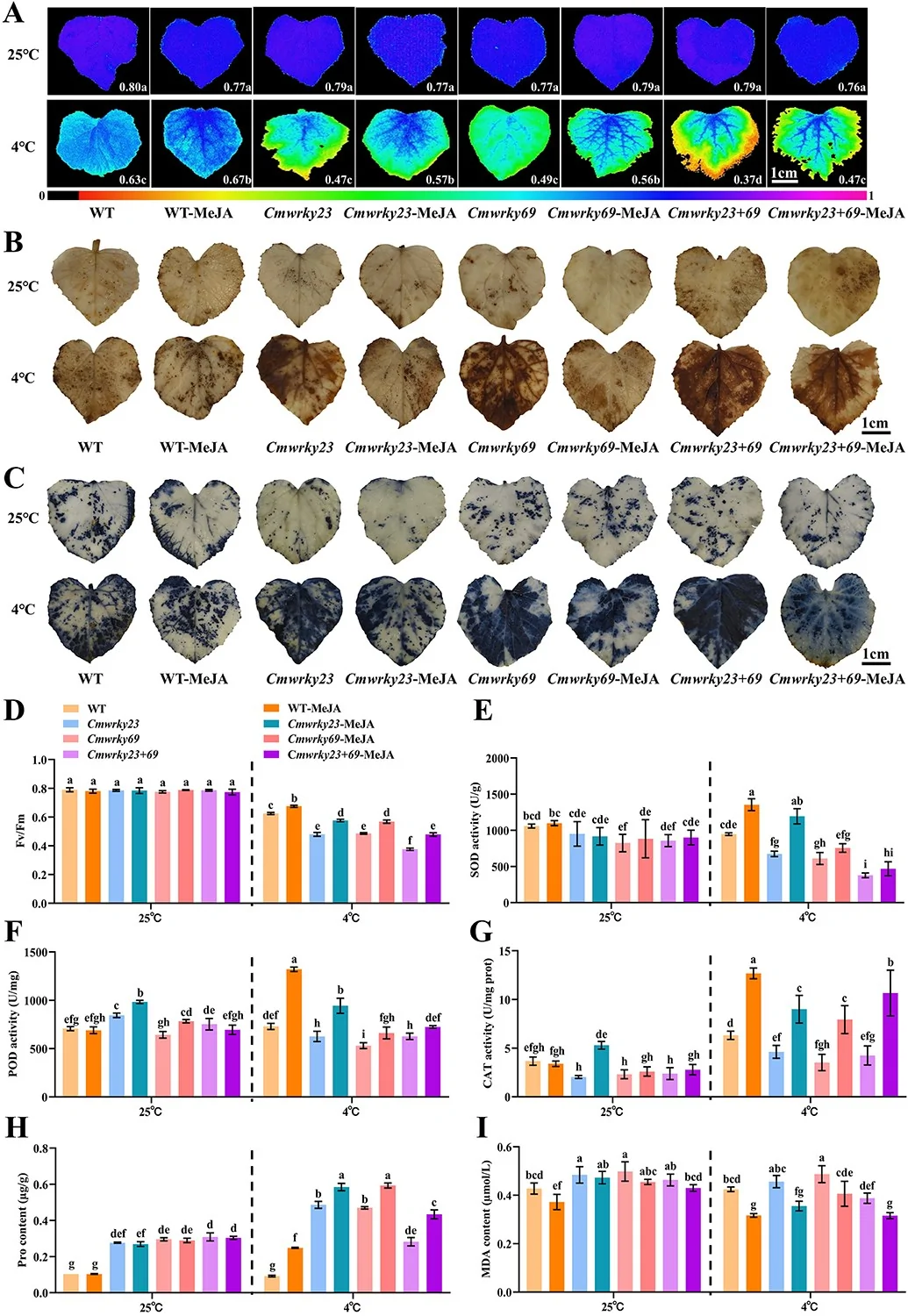
Exogenous MeJA affected the low-temperature tolerance in *Cmwrky23*, *Cmwrky69,* and *Cmwrky23*+*69*-knockout oriental melon plants. (A) Images of the maximum photochemical efficiency of PSII (Fv/Fm). The intensity scale at the bottom of the image ranges from 0 to 1. Different letters indicate significant differences at *P* < 0.05 (one-way ANOVA). Scale bar = 1 cm. (D) Fv/Fm ratio. (B and C) DAB and NBT staining. Scale bar = 1 cm. (E–G) SOD, POD, and CAT enzyme activity. (H and I) Pro, MDA content. Data represent mean ± SD (*n* = 3). Different letters indicate significant differences (ANOVA, *P* < 0.05).

## Discussion

The graft union is a diverse biological process that encompasses wound response, cellular dedifferentiation, redifferentiation, and vascular reconnection [18]. For grafted melon seedlings to survive and grow, both successful graft union formation and abiotic stress tolerance are essential, where plant hormones serve as key regulators [19, 20]. RNA-seq analysis of hickory graft healing identified 112 genes with altered expression linked to auxin and cytokinin signaling [21]. Within 1 day postgrafting, Yin *et al.* reported that IAA-related gene transcript levels in *Arabidopsis thaliana* grafts rose markedly. Jasmonic acids (JAs), including jasmonic acid (JA) and its methyl ester (methyl jasmonic acid, MeJA), are important phytohormones that contribute to the graft union healing process [22, 23]. Grafting induces a wound stress response in both the scion and rootstock at the cut graft interface [24]. Among them, JAs accumulate in large quantities at the injury site and activate a series of defense responses [25]. By triggering a wound stress response, exogenous MeJA helps grafted seedlings transition to the isolation layer stage and accelerates tissue formation during graft union healing [26]. We experimentally confirmed that exogenous MeJA treatment of oriental melon scions could induce upregulation of CmWRKY23, CmWRKY69, and *CmGH9B3* expression, thereby accelerating graft union healing (Fig. 1C–E). This process can be evaluated by acid fuchsin absorption and paraffin sectioning (Fig. 1A and B).

Prior research has shown that MeJA can improve cold tolerance in a variety of economically important crops [27]. MeJA effectively alleviates cold damage by reducing the water loss index, electrolyte leakage, and MDA accumulation in green pepper cells [28]. JA is produced by chloroplast membranes, which, in turn, is catalyzed by JAR1, AOS, OPR3, LOX2, and AOC2, and plays a key role in balancing plant development with stress adaptation. As a key enzyme in jasmonic acid (JA) biosynthesis, oxidative propadiene cyclase (AOC) is closely associated with plant growth and development and helps plants cope with environmental stress [29]. In alfalfa, boosting *MtAOC2* expression increases JA production and upregulates CBF, a cold-responsive gene, thereby improving cold tolerance [13]. Nevertheless, how JA mediates cold-stress responses in grafted melon seedlings remains unknown at the molecular level. Our results showed that exogenous MeJA application significantly upregulated *CmAOC2* expression during graft union healing in oriental melon grafted onto squash rootstocks. The relative expression exhibited a significant upregulation by low-temperature stress, indicating that it might serve as a potential regulator of the grafted oriental melon seedlings’ response to low-temperature stress (Fig. 5B).

Transcriptional regulation is a crucial process in all organisms, enabling gene expression to be tuned according to internal or external signals [30]. Up to now, fewer WRKYs have been identified as major cold stress regulators in plants. By way of example, in tomato (*S. lycopersicum* L.), SlWRKY50 and JA signaling-mediated positive feedback pathways are positively regulated by cold stress [16]. In bermudagrass [*Cynodon dactylon* (L.) Pers.], CdWRKY2 binds to the *CdCBF1* promoter to enhance cold resistance [31]. According to Guo *et al.*, tomato cold resistance is strongly boosted by ShWRKY33, which possesses auto-transcriptional activity [32]. In our study, oriental melon plants silenced for CmWRKY23 and CmWRKY69 showed a significant decrease in proline content and SOD activity, and an increase in MDA content during plant chilling stress (Fig. 7A–F). The positive roles of CmWRKY23 and CmWRKY69 in oriental melon’s low-temperature response were further confirmed. To comprehensively explore the roles of CmWRKY23 and CmWRKY69 in the low-temperature tolerance of oriental melon seedlings, we also constructed CRISPR/Cas9-mediated silent transgenic lines, as well as single- and double-knockout mutants of CmWRKY23 and CmWRKY69; all of these mutants exhibited low-temperature tolerance (Fig. 8A). Meanwhile, WRKY can activate downstream gene expression via the targeting of the W-box motif in the promoter [33]. EMSA and Y1H showed both CmWRKY23 and CmWRKY69 to bind the *CmAOC2* promoter (Fig. 6C and E). Based on the results of LUC and GUS experiments, CmWRKY23 and CmWRKY69 can activate the *CmAOC2* promoter, respectively (Fig. 6D; S2A and B). Furthermore, CmWRKY23 and CmWRKY69 act as common upstream regulatory hubs and are significantly induced by exogenous MeJA (Fig. 6A and B). In the downstream regulatory pathway, they directly activate *CmGH9B3* to promote cell adhesion, callus proliferation, and vascular reconnection of the graft union healing process. On the other hand, they specifically bind to the promoter of *CmAOC2*, thereby enhancing JA biosynthesis, antioxidant enzyme activities, and reactive oxygen species (ROS) homeostasis. Taken together, these findings demonstrated that MeJA modulates graft union healing and cold tolerance through an interconnected regulatory network.

In summary, this study proposed a novel model to explain how MeJA synergistically promoted the graft union healing and low-temperature tolerance in oriental melon seedlings (Fig. 10). According to this model, exogenous MeJA acted as a trigger, initially activating the transcription factors CmWRKY23 and CmWRKY69 at the graft union site. These two factors subsequently initiated two parallel pathways: (1) direct activation of the cell adhesion gene *CmGH9B3*, accelerating physical adhesion at the graft interface and vascular bundle reconnection; and (2) activation of the key JA biosynthesis gene *CmAOC2*, initiating a WRKY-based positive feedback loop that leads to endogenous JA accumulation, thereby systemically enhancing the scion’s ROS scavenging capacity and conferring stronger low-temperature tolerance to the grafted oriental melon seedlings. This discovery not only elucidated the traditional wisdom that MeJA promotes graft union healing at the molecular level but also revealed a novel mechanism by which MeJA couples graft union healing with low-temperature tolerance through WRKY-mediated transcriptional cascades. The findings provided important theoretical support and precise molecular targets for enhancing both survival and quality of grafted oriental melon seedlings under protected cultivation.

**Figure 10:**
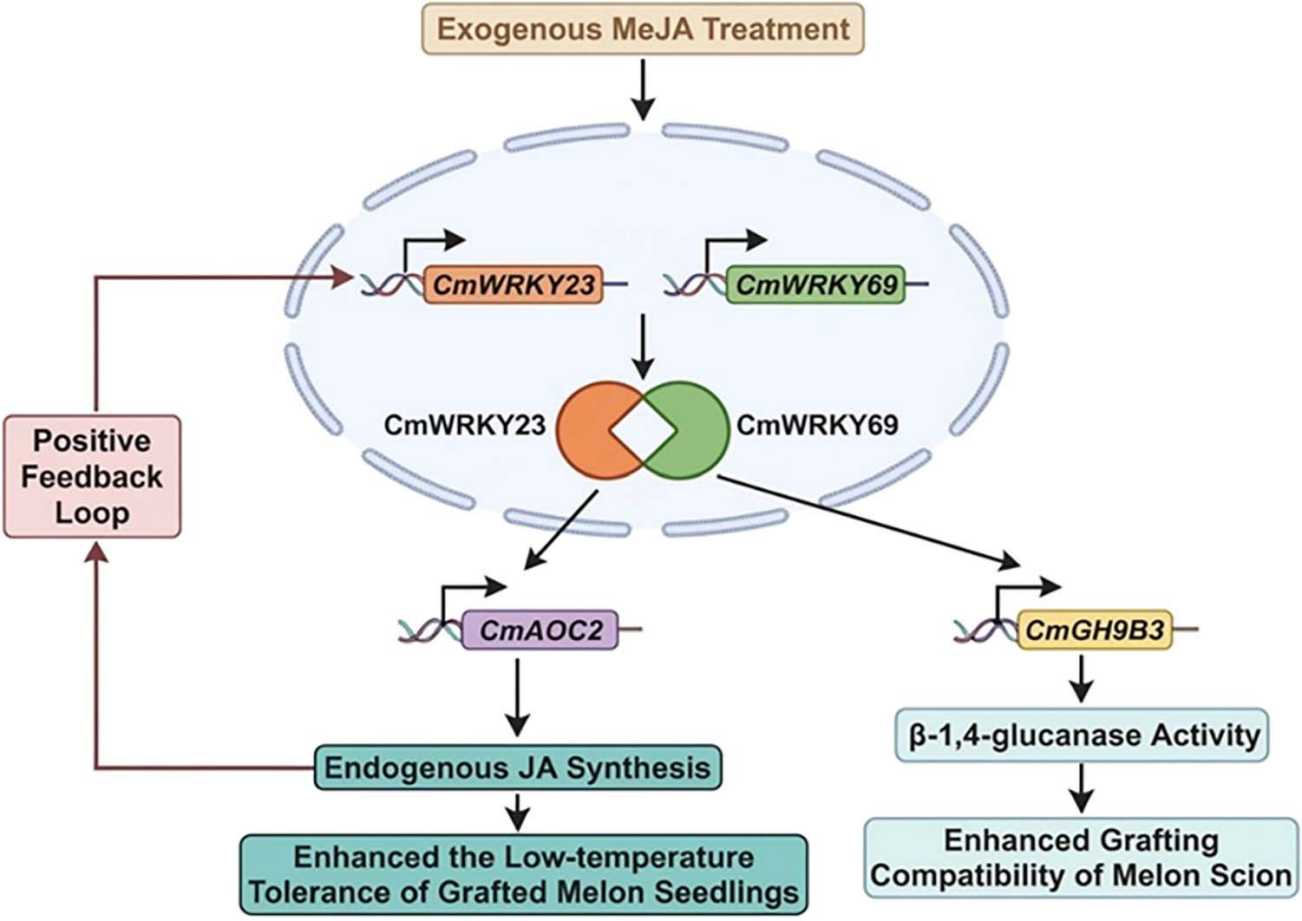
Molecular mechanism model of how exogenous methyl jasmonate (MeJA) treatment promotes graft union healing and low-temperature tolerance in the grafted oriental melon seedlings through dual regulatory modules. On the one hand, exogenous MeJA activated the CmWRKY23/CmWRKY69-*CmGH9B3* module, accelerating the healing of the graft union; on the other hand, it upregulated *CmAOC2* via CmWRKY23 and CmWRKY69, promoting jasmonic acid accumulation and forming a positive feedback loop, thereby enhancing the low-temperature tolerance of grafted oriental melon seedlings.

## Materials and methods

### Plant materials and grafting methods

This study used oriental melon ‘HuaBao’ (*C. melo* var. *makuwa* Makino) as scions and squash ‘ShengZhen NO.1’ (*Cucurbita moschata*) as rootstocks. At the cotyledon-unfolding stage, oriental melon leaves were sprayed three times with either 200 μM methyl jasmonate (MeJA) or 100 μM diethyldithiocarbamic acid (DIECA), with distilled water serving as the control. Grafting was performed using the one-cotyledon, one-leaf method once the oriental melon reached the one-leaf stage. Using a sharp grafting blade, a 45° diagonal cut on the scion stem was made, and part of the stem and roots was removed. One cotyledon and the growing point of the squash rootstock were removed. Then, the two wounds were attached together and fixed with a grafting clamp [34]. After grafting, seedlings were cultured under complete darkness for the first 2 days after grafting. Subsequently, the light duration was gradually increased according to the growth status of grafted seedlings until complete survival was achieved. At 2, 3, 4, 6, and 9 days after grafting (DAG), stem tissues were harvested from grafted seedlings that had identical growth traits. The seedlings were grown in a plant incubator during the day (12 h with 300 μmol photons·m^−2^·s^−1^ illumination at 4°C) and night (12 h darkness at 4°C). Throughout the experiment, the seedlings were grown under consistent environmental conditions. Samples were taken from leaf tissue after 72 h of low-temperature treatment. All samples were collected under identical environmental conditions. All collected samples were plunged into liquid nitrogen for freezing and subsequently preserved at −80°C. Three bio-replicates were established per treatment, and *N. benthamiana* employed in this study were cultivated in a growth chamber set to 25°C under a 16/8 h light/dark cycle.

### Histological section observation

Plant material was collected from stems within 0.5–1.0 cm on either side of the graft junction, with the rootstock and scion measuring 2.5–5 mm each. These samples were then processed through fixation, softening, dehydration, infiltration, and embedding in Leica paraffin, as per the methodology as reported earlier by Ribeiro *et al.* [35]. After slicing into transverse serial sections approximately 10 μm thick, samples were stained with saffron (solid green) and mounted in synthetic resin (Permount). Examination of the sections was conducted utilizing a light microscope (Leica RM 2245, Germany). The acid fuchsin uptake assay was performed to determine whether a vascular connection had been established between the scion and rootstock. Cross-cuts were made on 1.0-cm stem segments taken from both sides of the graft junction. Rootstock stems were immersed upright in a 1% acidic fuchsin solution for 1 h. Additionally, 2.5-mm stem pieces above the graft union were cross-sectioned for assessment of acid fuchsin uptake, and confocal laser microscopy was used for imaging. Three bio-replicates were prepared.

### RNA extraction, cDNA synthesis, and gene expression analysis

Total RNA was extracted from melon stem tissues via liquid nitrogen grinding and processed with the Ultrapure RNA Kit according to the manufacturer’s instructions. First-strand cDNA was generated from 1000 ng of total RNA using PrimeScript™ RT Master Mix (Takara, Dalian, China). Subsequent RT-qPCR assays used this cDNA as a template. The relative expression of JA biosynthesis key genes (*CmAOC2*, *CmJAR1*, *CmAOS*, *CmOPR3*, and *CmLOX2*) in oriental melon stems was measured via RT-qPCR. A Bio-Rad CFX96 PCR thermocycler and Pro Taq HS SYBR Green premix qPCR kit (AG) were used to conduct RT-qPCR in a 20-μl reaction system [36]. Three distinct biological experiments were carried out under identical conditions to reproduce the findings. Primer3 was used to design the RT-qPCR primers, and detailed primer sequences are listed in Table S1. The 2^−ΔΔCt^ method was used to determine transcript levels, using melon 18s rRNA as an internal reference. For every experiment, we set up three replicates at both the biological and technical levels.

### Subcellular localization

pCAMBIA1300 was used to express CmWRKY23 and CmWRKY69 as GFP fusion proteins from their full-length, stop-codon-free sequences, generating the reporter constructs 35S:: CmWRKY23-GFP and 35S:: CmWRKY69-GFP. Using an empty vector as a reference, we introduced the construct into *Agrobacterium tumefaciens* GV3101 and subsequently infiltrated it into *N. benthamiana* leaves. Following this, the leaves were stained with DAPI for 5 min. Subcellular localization was then assessed with a confocal laser scanning microscope (TCS SP8, Leica, Wetzlar, Germany) [37].

### DAB and NBT staining

Staining was performed using NBT (Sigma-Aldrich Co., St Louis, USA) and 3,30-diaminobenzidine (DAB, Sigma-Aldrich Co., St. Louis, USA), respectively [37]. For NBT staining, the plants were immersed in 50 mM phosphate buffer (pH 7.8) and placed on a 28°C shaker. Subsequently, they were destained with 95% ethanol and photographed. For DAB staining, samples were submerged in DAB solution (pH 3.8) and left under light for 6 h, followed by destaining with 95% alcohol and subsequent imaging.

### Virus-induced gene silencing assay

Using the gene-specific primers (Table S2), we PCR-amplified cDNA fragments of CmWRKY23 and CmWRKY69 to generate virus-induced gene silencing melon plants. The control was an empty pTRV2 vector. Each of these constructs was transformed separately into *A. tumefaciens* GV3101, and melon plants were then transformed following the method of Ming *et al.* [29]. Fully expanded leaves were sampled at 14 days and examined via RT-qPCR to identify silenced plants.

### Yeast one-hybrid assay

The *CmAOC2* promoter was cloned into the pAbAi vector (*CmAOC2pro*-pAbAi). The complete coding sequences of CmWRKY23 and CmWRKY69 were ligated into the pGADT7 vector (CmWRKY23-pGADT7, CmWRKY69-pGADT7). We transformed the vectors *CmAOC2pro*-pAbAi, CmWRKY23-AD, and CmWRKY69-AD, which were then co-expressed in yeast cells. The control group consisted of the compound containing *CmAOC2pro*-pAbAi and the pGADT7 (AD) vector. This assay employed Aureobasidin A (AbA, Warbio, China) at five concentrations, 50, 75, 100, 150, and 200 ng/ml. The experimental procedure was performed as previously reported [38].

### Luciferase reporter assay

The *CmAOC2* promoter was placed into the pGreenII 0800-LUC reporter vector, whereas the complete CDSs of CmWRKY23 and CmWRKY69 were inserted into the pCAMBIA1300-35S-GFP effector vector. *A. tumefaciens* strain GV3101 was transformed with the reporter and effector vectors. Following the method of An *et al.*, the *A. tumefaciens*-mediated infiltration of *N. benthamiana* leaves was performed using needleless syringes [39]. Different sides of the vein were infiltrated by the interaction group and the control group, respectively. Three days later, fluorescence imaging of the infiltrated *N. benthamiana* leaves was performed using a live imager (Lb985, Berthold, Germany).

### GUS assay

CmWRKY23 and CmWRKY69 full-length CDSs were cloned into pRI101, yielding the effector construct. Utilizing the *CmAOC2* promoter sequence, the reporter constructs were developed by integrating it upstream of the GUS reporter gene within the pBI101 vector. *N. benthamiana* leaves were co-infiltrated with *A. tumefaciens* GV3101 harboring both reorter and effector constructs. GUS activity was evaluated according to the protocol of Lv *et al.* [38].

### Electrophoretic mobility shift assay

After cloning into the pD2P-1.06eTM vector, the CmWRKY23 and CmWRKY69 CDSs were used to generate CmWRKY23-His and CmWRKY69-GST fusion proteins, respectively. Sebastian (Beijing, China) Biological Company synthesized the probes. Double-stranded DNA probes with 3′-biotin end labeling were created by annealing complementary oligonucleotides for EMSA. For the binding assay, we used the Light Shift Chemiluminescent EMSA Kit (Cat. no. 20148, Thermo Scientific), in accordance with the procedure described by Liu *et al.* [40].

### Stable transformation of oriental melon

Generation of the CmWRKY23 and CmWRKY69 knockout mutants was achieved through the CRISPR-Cas9 system, with the design of two targets facilitated by CRISPR (https://www.addgene.org/crispr/). Insertion of the gRNAs into the pHSE402 vector was followed by transformation of the correct recombinant plasmid into *Agrobacterium* strain EHA105 using oriental melon cotyledon. The WT ‘T0948’ was designated as the reference. Transgenic lines were identified using specific primers (Table S3), and aada-resistant plants were then selected for further examination. After inserting the gRNAs into the pHSE402 vector, the correct recombinant plasmid was transformed into *Agrobacterium* strain EHA105 via oriental melon cotyledons. The WT ‘T0948’ was utilized. Transgenic lines were identified using specific primers (Table S3), and plants resistant to aada were selected for subsequent analysis.

### Measurement of physiological indicators

Plant height, scion stem thickness, and pitch of knots were measured using digital vernier calipers with an accuracy of 0.01 cm. The plants were initially dried at 105°C for 30 min, then at 80°C until a stable weight was achieved. An electronic balance (0.0001 g precision) was used to determine fresh weight and dry matter content. Evaluation of superoxide dismutase (SOD) activity was carried out employing the NBT method [41]. Peroxidase (POD) activity was measured by the guaiacol method [42]. Catalase (CAT) activity was assessed via UV spectrophotometry, with 1 U defined as a 0.01 reduction in absorbance per minute at 240 nm [43]. Proline (Pro) content was measured as described by Yuan *et al.* [44]. The determination of MDA content was conducted utilizing the thiobarbituric acid (TBA) method [45].

### Quantification of JA contents

JA was quantified using ELISA detection kits (FY7957-A, Shanghai, China). Per 100 mg of tissue, homogenization was done in 1 ml of prechilled phosphate buffer (pH 7.8). Absorbance measurements at 450 nm were obtained with a microplate reader (Tecan), in line with the protocol described by Ming *et al.* [29].

### Statistical analysis

The data were analyzed utilizing SPSS V26.0 to determine the mean, standard deviation, and significant variances among samples. Data are shown as means ± SD. Statistical significance was evaluated by one-way ANOVA combined with Duncan’s multiple range test (*P <* 0.05) and independent t-tests, with asterisks marking significant differences (^*^P < 0.05, ^**^P < 0.01).

## Supplementary Material

Web_Material_uhag220

## Data Availability

The data that support the findings of this study are available in the manuscript and Supporting Information of this article. The authors confirm that all experimental data are available and can be accessed through the main text and/or the supplemental data. The accession codes are listed as follows: *CmGH9B3* (MELO3C022160), CmWRKY23 (MELO3C015910), CmWRKY69 (MELO3C018717), *CmCBF1* (MELO3C006869), *CmMYC2* (MELO3C003412), *CmJAZ2* (MELO3C024336), *CmAOS* (MELO3C010910), *CmLOX2* (MELO3C014627), *CmOPR3* (MELO3C029299), *CmAOC2* (MELO3C003015), and *CmJAR1* (MELO3C006046).

## References

[1] Kyriacou MC, Rouphael Y, Colla G. et al. Vegetable grafting: the implications of a growing agronomic imperative for vegetable fruit quality and nutritive value. Front Plant Sci. 2017;8:741 10.3389/fpls.2017.00741 PMC5427113 28553298

[2] Bantis F, Koukounaras A, Siomos AS. et al. Bichromatic red and blue LEDs during healing enhance the vegetative growth and quality of grafted watermelon seedlings. Sci Hortic. 2020;261:109000

[3] Abouseif Y, Kadeer A, Cao H. et al. Integrated multi-omics analysis reveals the molecular mechanism of light intensity-enhanced healing in cotyledon-less splice grafted watermelon. Hortic Res. 2026;13:uhaf293 10.1093/hr/uhaf293 PMC12923272 41726326

[4] Chinnusamy V, Zhu J, Zhu JK. Cold stress regulation of gene expression in plants. Trends Plant Sci. 2007;12:444–51 10.1016/j.tplants.2007.07.002 17855156

[5] Korkmaz A, Dufault RJ. Influence of short-term cyclic cold temperature stress on muskmelon and honeydew yield. HortTechnology. 2003;13:67–70

[6] Li H, Guo Y, Lan Z. et al. Methyl jasmonate mediates melatonin-induced cold tolerance of grafted watermelon plants. Hortic Res. 2021;8:57 10.1038/s41438-021-00496-0 PMC7943586 33750773

[7] Wang L, Wu B, Chen G. et al. The essential role of jasmonate signaling in *Solanum habrochaites* rootstock-mediated cold tolerance in tomato grafts. Hortic Res. 2023;10:uhac227 10.1093/hr/uhac227 PMC9832872 36643752

[8] Yang Z, Li G, Zhang Y. et al. Crop antiviral defense: past and future perspective. Sci China Life Sci. 2024;67:2617–34 10.1007/s11427-024-2680-3 39190125

[9] Shi F, Ni Y, Qiu Y. et al. Changes in phytohormone and antioxidant enzyme activities during the healing process of different crabapple rootstock–scion combinations. Forests. 2023;14:1362

[10] Ntatsi G, Savvas D, Papasotiropoulos V. et al. Rootstock sub-optimal temperature tolerance determines transcriptomic responses after long-term root cooling in rootstocks and scions of grafted tomato plants. Front Plant Sci. 2017;8:911 10.3389/fpls.2017.00911 PMC5462977 28642763

[11] Hu Y, Jiang L, Wang F. et al. Jasmonate regulates the inducer of CBF expression–c-repeat binding factor/DRE binding factor1 cascade and freezing tolerance in *Arabidopsis*. Plant Cell. 2013;25:2907–24 10.1105/tpc.113.112631 PMC3784588 23933884

[12] Yu X, Zhang W, Zhang Y. et al. The roles of methyl jasmonate to stress in plants. Funct Plant Biol. 2019;46:197–212 10.1071/FP18106 32172764

[13] Yang L, Sun Q, Geng B. et al. Jasmonate biosynthesis enzyme allene oxide cyclase 2 mediates cold tolerance and pathogen resistance. Plant Physiol. 2023;193:1621–34 10.1093/plphys/kiad362 37392433

[14] Sharma M, Laxmi A. Jasmonates: emerging players in controlling temperature stress tolerance. Front Plant Sci. 2016;6:1129 10.3389/fpls.2015.01129 PMC4701901 26779205

[15] Zhu Y, Guo J, Wu F. et al. Genome-wide characteristics of GH9B family members in melon and their expression profiles under exogenous hormone and far-red light treatment during the grafting healing process. Int J Mol Sci. 2023;24:8258 10.3390/ijms24098258 PMC10179234 37175962

[16] Wang L, Chen H, Chen G. et al. Transcription factor SlWRKY50 enhances cold tolerance in tomato by activating the jasmonic acid signaling. Plant Physiol. 2024;194:1075–90 10.1093/plphys/kiad578 37935624

[17] Zhu Y, Li J, Wu H. et al. CmHY5-CmWRKY23/69-*CmGH9B3* module mediates red light promoted graft union healing of melon grafted onto squash. Hortic Res. 2026;13:uhaf251 10.1093/hr/uhaf251 PMC12858254 41624422

[18] Tedesco S, Fevereiro P, Kragler F. et al. Plant grafting and graft incompatibility: a review from the grapevine perspective. Sci Hortic. 2022;299:111019

[19] Ohyanagi H, Nakamura Y, Yano K. *Plant and Cell Physiology's* 2018 database issue and beyond. Plant Cell Physiol. 2018;59:1–2 10.1093/pcp/pcy002 29931342

[20] Xu C, Zhang Y, Zhao M. et al. Transcriptomic analysis of melon/squash graft junction reveals molecular mechanisms potentially underlying the graft union development. PeerJ. 2021;9:e12569 10.7717/peerj.12569 PMC8675255 34993019

[21] Qiu L, Jiang B, Fang J. et al. Analysis of transcriptome in hickory (*Carya cathayensis*), and uncover the dynamics in the hormonal signaling pathway during graft process. BMC Genomics. 2016;17:935 10.1186/s12864-016-3182-4 PMC5114764 27855649

[22] Yin H, Yan B, Sun J. et al. Graft-union development: a delicate process that involves cell–cell communication between scion and stock for local auxin accumulation. J Exp Bot. 2012;63:4219–32 10.1093/jxb/ers109 PMC3398452 22511803

[23] Huang H, Liu B, Liu L. et al. Jasmonate action in plant growth and development. J Exp Bot. 2017;68:1349–59 10.1093/jxb/erw495 28158849

[24] Sheard LB, Tan X, Mao H. et al. Jasmonate perception by inositol-phosphate-potentiated COI1–JAZ co-receptor. Nature. 2010;468:400–5 10.1038/nature09430 PMC2988090 20927106

[25] Li C, Xu M, Cai X. et al. Jasmonate signaling pathway modulates plant defense, growth, and their trade-offs. Int J Mol Sci. 2022;23:3945 10.3390/ijms23073945 PMC8999811 35409303

[26] Ikeuchi M, Iwase A, Rymen B. et al. Wounding triggers callus formation via dynamic hormonal and transcriptional changes. Plant Physiol. 2017;175:1158–74 10.1104/pp.17.01035 PMC5664475 28904073

[27] Zhao M, Wang J, Shan W. et al. Induction of jasmonate signaling regulators MaMYC2s and their physical interactions with MaICE1 in methyl jasmonate-induced chilling tolerance in banana fruit. Plant Cell Environ. 2013;36:30–51 10.1111/j.1365-3040.2012.02551.x 22651394

[28] Ma M, Zhu Z, Cheng S. et al. Methyl jasmonate alleviates chilling injury by regulating membrane lipid composition in green bell pepper. Sci Hortic. 2020;266:109308

[29] Ming R, Zhang Y, Wang Y. et al. The JA-responsive MYC2-BADH-like transcriptional regulatory module in *Poncirus trifoliata* contributes to cold tolerance by modulation of glycine betaine biosynthesis. New Phytol. 2021;229:2730–50 10.1111/nph.17063 33131086

[30] Wani SH, Anand S, Singh B. et al. WRKY transcription factors and plant defense responses: latest discoveries and future prospects. Plant Cell Rep. 2021;40:1071–85 10.1007/s00299-021-02691-8 33860345

[31] Huang X, Cao L, Fan J. et al. CdWRKY2-mediated sucrose biosynthesis and CBF-signaling pathways coordinately contribute to cold tolerance in bermudagrass. Plant Biotechnol J. 2022;20:660–75 10.1111/pbi.13745 PMC8989505 34743386

[32] Guo M, Yang F, Liu C. et al. A single-nucleotide polymorphism in WRKY33 promoter is associated with the cold sensitivity in cultivated tomato. New Phytol. 2022;236:989–1005 10.1111/nph.18403 35892173

[33] Ciolkowski I, Wanke D, Birkenbihl RP. et al. Studies on DNA-binding selectivity of WRKY transcription factors lend structural clues into WRKY-domain function. Plant Mol Biol. 2008;68:81–92 10.1007/s11103-008-9353-1 PMC2493524 18523729

[34] Zhu Y, Guo J, Wu F. et al. Exogenous melatonin application accelerated the healing process of oriental melon grafted onto squash by promoting lignin accumulation. Int J Mol Sci. 2024;25:3690 10.3390/ijms25073690 PMC11011509 38612499

[35] Ribeiro L, Nery L, Vieira L. et al. Histological study of micrografting in passionfruit. Plant Cell Tiss Org Cult. 2015;123:173–81

[36] Hou S, Zhu Y, Wu X. et al. Scion-to-rootstock mobile transcription factor CmHY5 positively modulates the nitrate uptake capacity of melon scion grafted on squash rootstock. Int J Mol Sci. 2022;24:162 10.3390/ijms24010162 PMC9820822 36613606

[37] Wu TM, Huang JZ, Oung HM. et al. H_2_O_2_-based method for rapid detection of transgene-free rice plants from segregating CRISPR/Cas9 genome-edited progenies. Int J Mol Sci. 2019;20:3885 10.3390/ijms20163885 PMC6720670 31404948

[38] Lv B, Wu Q, Wang A. et al. A WRKY transcription factor, FtWRKY46, from Tartary buckwheat improves salt tolerance in transgenic *Arabidopsis thaliana*. Plant Physiol Biochem. 2021;147:43–53 10.1016/j.plaphy.2019.12.004 31841961

[39] An JP, Wang XF, Zhang XW. et al. Apple B-box protein BBX37 regulates jasmonic acid mediated cold tolerance through the JAZ-BBX37-ICE1-CBF pathway and undergoes MIEL1-mediated ubiquitination and degradation. New Phytol. 2021;229:2707–29 10.1111/nph.17050 33119890

[40] Liu Y, Du M, Deng L. et al. MYC2 regulates the termination of jasmonate signaling via an autoregulatory negative feedback loop. Plant Cell. 2021;31:106–27 10.1105/tpc.18.00405 PMC6391702 30610166

[41] Singh O, Tyagi N, Olmstead MM. et al. The design of synthetic superoxide dismutase mimetics: seven-coordinate water soluble manganese (II) and iron (II) complexes and their superoxide dismutase-like activity studies. Dalton Trans. 2017;46:14186–91 10.1039/c7dt03278a 28984335

[42] Marchand L, Grebenshchykova Z, Mench M. Intra-specific variability of the guaiacol peroxidase (GPOD) activity in roots of Phragmites australis exposed to copper excess. Ecol Eng. 2016;90:57–62

[43] Wang N, Zhang W, Qin M. et al. Drought tolerance conferred in soybean (*Glycine max* L.) by GmMYB84, a novel R2R3-MYB transcription factor. Plant Cell Physiol. 2017;58:1764–76 10.1093/pcp/pcx111 29016915

[44] Yuan HM, Sheng Y, Chen WJ. et al. Overexpression of Hevea brasiliensis HbICE1 enhances cold tolerance in *Arabidopsis*. Front Plant Sci. 2017;8:1462 10.3389/fpls.2017.01462 PMC5572258 28878797

[45] Cai W, Liu W, Wang WS. et al. Overexpression of rat neurons nitric oxide synthase in rice enhances drought and salt tolerance. PLoS One. 2015;10:e0131599 10.1371/journal.pone.0131599 PMC4485468 26121399

