# Supplementary material for: Exogenous methyl jasmonate promotes the graft union healing process of melon grafted onto squash and enhances low-temperature tolerance of grafted melon seedlings by activating the transcription factors CmWRKY23 and CmWRKY69
Source: Hortic Res. 2026 Jun 3;13(11):uhag220. doi: 10.1093/hr/uhag220 (PMC13722867; doi:10.1093/hr/uhag220)
Supplement: Supplementary file 1 [file web_material_uhag220.zip › Supplementary Figure.docx]

**Figure S1.** Overexpression of *CmAOC2* reduces the low-temperature sensitivity of oriental melon seedlings. (A) Images of the maximum photochemical efficiency of PSII (Fv/Fm). The false-color code at the bottom of the image ranges from 0 to 1. Different letters indicate significant differences at *p* < 0.05 (1-way ANOVA). Scale bar = 1 cm. (B) DAB and NBT staining. Scale bar = 1 cm. (C) Expression level of *CmAOC2* in the OE plants under low-temperature stress. (D) Fv/Fm ratio. (E) Endogenous JA content. (F) SOD activity. (G) POD activity. (H) CAT activity. (I) Pro activity. (J) MDA content. Data represent mean ± SD (*n* = 3). Different letters indicate significant differences (ANOVA, *p* < 0.05).

**Figure S2.** GUS activity assays analysis of CmWRKY23 and CmWRKY69 binding to the *CmAOC2* promoter in *N. benthamiana* leaves. (A, B) GUS activity assays analysis of CmWRKY23 and CmWRKY69 separately binding to the *CmAOC2* promoter in *N. benthamiana* leaves. An increase in relative GUS activity indicates that transcriptional regulation is promoted. An independent *t*-test was used to analyze the difference between the treatment and control groups. The data are the means of 3 replicates (±SD). *n* = 3. Statistical significance levels (Student’s *t*-test) were shown: ns; **p* ≤ 0.05; ***p* ≤ 0.01; ****p* ≤ 0.001; *****p* ≤ 0.0001.

**Figure S3.** Effects of silencing CmWRKY23 and CmWRKY69 on their gene expression, and physiological indices under low-temperature stress. (A, B) Relative expression of CmWRKY23 and CmWRKY69 in oriental melon stems after transient silencing of CmWRKY23 and CmWRKY69. (C) Plant height. (D) Stem thickness. (E) Pitch of knots. (F) Dry weight above ground. (G) Fresh weight above ground. (H) Sub-ground dry weight. (I) Sub-ground fresh weight. TRV: Empty vector-infected oriental melon plants.TRV-CmWRKY23: Transient silencing of CmWRKY23 in infected oriental melon plants.TRV-CmWRKY69: Transient silencing of CmWRKY69 infected oriental melon plants.TRV-CmWRKY23+CmWRKY69: Transient silencing of TRV-CmWRKY23+CmWRKY69 co-infected oriental melon plants. Different letters indicate significant differences (ANOVA, *p*<0.05). Values are means ± SD, *n* = 3.

**Figure S4.** Overexpression of the CmWRKY23 and CmWRKY69 alleviated low-temperature sensitivity in oriental melon seedlings. (A-C) Expression analysis of CmWRKY23, CmWRKY69, and *CmAOC2* among the WT and CmWRKY23-OE, CmWRKY69-OE, and CmWRKY23+69-OE of oriental melon seedlings under 4℃ low-temperature treatment. (D) Images of the maximum photochemical efficiency of PSII (Fv/Fm). The false-color code at the bottom of the image ranges from 0 to 1. Different letters indicate significant differences at *p* < 0.05. Scale bar = 1 cm. (E) Fv/Fm. (F, G) DAB and NBT staining. (H) Endogenous JA content. (I-K) SOD, POD, and CAT enzyme activity. (L, M) Pro and MDA content. Data represent mean ± SD (*n* = 3). Different letters indicate significant differences (ANOVA, *p* < 0.05).
