# Supplementary material for: Exogenous methyl jasmonate promotes the graft union healing process of melon grafted onto squash and enhances low-temperature tolerance of grafted melon seedlings by activating the transcription factors CmWRKY23 and CmWRKY69
Source: Hortic Res. 2026 Jun 3;13(11):uhag220. doi: 10.1093/hr/uhag220 (PMC13722867; doi:10.1093/hr/uhag220)
Supplement: Supplementary file 1 [file web_material_uhag220.zip › Supplementary Table.docx]

**Table S1.** Genes and primers used in RT‐qPCR

| **Accession number** | **Primer name** | **Primer sequence** |
| --- | --- | --- |
| MELO3C015910 | CmWRKY23-F | AATGGCCTACCACGCCAAAT |
|  | CmWRKY23-R | CGAATCGTGGTTCCACACCT |
| MELO3C018717 | CmWRKY69-F | ATGACTCAAAACCCACCGCT |
|  | CmWRKY69-R | CTCCATGCCCATGAATCGGA |
| MELO3C010910 | *CmAOS*-F | GGTTCAGAACAAGCAGTGCG |
|  | *CmAOS-R* | *TCTTTAGGGAGGTTACGGTGA* |
| MELO3C014627 | *CmLOX*2-F | TTTTGGAGCTCGTCAGTGCT |
|  | *CmLOX2*-R | GGAGGCCTTCGATGACAACA |
| MELO3C029299 | *CmOPR3*-F | TGCGGGAATACGCTAACCAT |
|  | *CmOPR3*-R | TTGGTCGGGTACGGCTAAAC |
| MELO3C003015 | *CmAOC2*-F | TCTACTTCGGCGATTACGGC |
|  | *CmAOC2*-R | ACCCGCAATCCCCTTCAAAT |
| MELO3C006046 | *CmJAR1*-F | TGCTGTCGTGATTCTCGAGTT |
|  | *CmJAR1*-R | GCAGCCAGACCACCATTAGT |
| MELO3C006869 | *CmCBF1*-F | AGAGTAGTAACTGCCGTCGC |
|  | *CmCBF1*-R | ACTTCACAGACCCACTTGCC |
| MELO3C003412 | *CmMYC2*-F | TGAGGTTGTTACCGACACCG |
|  | *CmMYC2*-R | CGAAAACCTGACCCTGACGA |
| MELO3C024336 | *CmJAZ2-*F | GGCGAGAGATCTTACTCGAGATTT |
|  | *CmJAZ2*-R | AACGAACTTCGGCAGACCAT |
| 18S | 18S-F | GTGATGGTGTGAGTCACACTGTTC |
| 18S | 18S-R | ACGACCAGCAAGGTCCAAAC |

**Table S2.** Gene amplification primer sequences

| **Accession number** | **Primer name** | **Primer sequence** |
| --- | --- | --- |
| MELO3C015910 | CmWRKY23-TRV2-F | GTGAGTAAGGTTACCGAATTC TTCTTTGATTTCAAATCCTGAG |
|  | CmWRKY23-TRV2-R | CGTGAGCTCGGTACCGGATCC TTTTGACCATACTTTCTCCATC |
| MELO3C018717 | CmWRKY69-TRV2-F | GTGAGTAAGGTTACCGAATTC TTCCAAGGGATGCCCAG |
|  | CmWRKY69-TRV2-R | CGTGAGCTCGGTACCGGATCC GGCTGACTCGCGAAAACC |
| MELO3C015910 | CmWRKY23-AD-F | GCCATGGAGGCCAGTGAATTC ATGGAGGAGGTGGTGGC |
|  | CmWRKY23-AD-R | CAGCTCGAGCTCGATGGATCC TTAATGGTGGCACCAAGC |
| MELO3C018717 | CmWRKY69-AD-F | GCCATGGAGGCCAGTGAATTC ATGGACCGCCGTGTTCGT |
|  | CmWRKY69-AD-R | CAGCTCGAGCTCGATGGATCC CTAGCAGTCTAAAACTGTGCCACAT |
| MELO3C003015 | *CmAOC2*-pABAI-F | GAAAAGCTTGAATTCGAGCTC GAAATGTGGCATGTGCAAC |
|  | *CmAOC2*-pABAI-R | ATACAGAGCACATGCCTCGAG CAAACCAAAGTCAGCAGAGAG |
| MELO3C015910 | CmWRKY23-PRI101-F | TCTTCACTGTTATACATATG ATGGAGGAGGTGGTGGC |
|  | CmWRKY23-PRI101-R | AGAGTTGTTGATTCAGAATTC TTAATGGTGGCACCAAGC |
| MELO3C018717 | CmWRKY69-PRI101-F | TCTTCACTGTTATACATATG ATGGACCGCCGTGTTCGT |
|  | CmWRKY69-PRI101-R | AGAGTTGTTGATTCAGAATTC CTAGCAGTCTAAAACTGTGCCACAT |
| MELO3C003015 | *CmAOC2*-PBI101-F | CTTGCATGCCTGTAGGTCGA GAAATGTGGCATGTGCAAC |
|  | *CmAOC2*-PBI101-R | AAGGGACTGACCACCC CAAACCAAAGTCAGCAGAGAG |
| MELO3C015910 | CmWRKY23-1300-F | GAGCTCGGTACCCGGGGATCC ATGGAGGAGGTGGTGGC |
|  | CmWRKY23-1300-R | GCCCTTGCTCACCATGTCGAC TTAATGGTGGCACCAAGC |
| MELO3C018717 | CmWRKY69-1300-F | GAGCTCGGTACCCGGGGATCC ATGGACCGCCGTGTTCGT |
|  | CmWRKY69-1300-R | GCCCTTGCTCACCATGTCGAC CTAGCAGTCTAAAACTGTGCCACAT |
| MELO3C003015 | *CmAOC2*-0800-F | GTCGACGGTATCGATAAGCTT GAAATGTGGCATGTGCAAC |
|  | *CmAOC2*-0800-R | CGCTCTAGAACTAGTGGATCC CAAACCAAAGTCAGCAGAGAG |
| MELO3C015910 | CmWRKY23-HIS-F | GCCATGGCTGATATCGGATCC ATGGAGGAGGTGGTGGC |
|  | CmWRKY23-HIS-R | CTCGAGTGCGGCCGCAAGCTT TTAATGGTGGCACCAAGC |
| MELO3C018717 | CmWRKY69-GST-F | TTCCAGGGGCCCCTGGGATCC ATGGACCGCCGTGTTCGT |
|  | CmWRKY69-GST-R | GTCACGATGCGGCCGCTCGAG CTAGCAGTCTAAAACTGTGCCACAT |

**Table S3.** Primers for detecting mutant plants

| **Primer name** | **Primer sequence** |
| --- | --- |
| *Cmwrky23*-F | CCAATTCTTCTTCACCCTCTTCTC |
| *Cmwrky23*-R | CCAAATCCAAAACCCTCTCCTGC |
| *Cmwrky69*-F | CCCAGCCCTACGATTCATCTTC |
| *Cmwrky69*-R | GCAATTTCCCCTTTGATTAAAGCTC |
| *Cmwrky23+69-23*-F | CCAATTCTTCTTCACCCTCTTCTC |
| *Cmwrky23+69-23*-R | CCAAATCCAAAACCCTCTCCTGC |
| *Cmwrky23+69-69*-F | CATTCATCACAACAATCTCCAGC |
| *Cmwrky23+69-69*-R | GCCGAAAATGTCCTTGAGCATCG |
